# Organ‐specific redox imbalances in spinal muscular atrophy mice are partially rescued by SMN antisense oligonucleotides

**DOI:** 10.1002/1873-3468.70303

**Published:** 2026-02-13

**Authors:** Sofia Vrettou, Brunhilde Wirth

**Affiliations:** ^1^ Institute of Human Genetics University Hospital of Cologne, University of Cologne Germany; ^2^ Center for Molecular Medicine Cologne University of Cologne Germany; ^3^ Center for Rare Diseases University Hospital of Cologne, University of Cologne Germany

**Keywords:** antisense oligonucleotides, GPX4, lipid peroxidation, redox enzymes, S‐glutathionylation, Spinal muscular atrophy

## Abstract

Spinal muscular atrophy (SMA) is caused by a deficiency in survival motor neuron (SMN) protein; redox imbalance and oxidative stress are also implicated. Protein S‐glutathionylation (PSSG) is a reversible redox modification that protects cysteines from irreversible oxidation and regulates protein function. Here, we report stage‐ and tissue‐dependent defects in PSSG levels, accompanied by tissue‐specific alterations in the expression of glutathione‐related enzymes in Taiwanese SMA mice at early and late symptomatic stages. Importantly, we also provide evidence linking glutathione homeostasis defects with ferroptosis. Finally, partial restoration of SMN by antisense oligonucleotides selectively modulates these abnormalities in a tissue‐dependent manner. Our findings suggest S‐glutathionylation dysregulation as a novel SMA hallmark and highlight persistent redox imbalance as a therapeutic target beyond SMN restoration.

**Impact statement**

This study provides a multi‐organ analysis of redox imbalance in spinal muscular atrophy, revealing systemic loss of protein S‐glutathionylation in a stage‐ and tissue‐dependent manner. By identifying the heart as particularly redox‐vulnerable, this work refines understanding of oxidative stress beyond motor neurons and informs tissue‐aware therapeutic evaluation.

## Graphical abstract

We identified a systemic, progressive loss of protein S‐glutathionylation—detected by nonreducing western blotting—alongside dysregulation of glutathione‐cycle enzymes in both neuronal and peripheral tissues of Taiwanese SMA mice. These alterations were partially rescued by SMN antisense oligonucleotide therapy, revealing persistent redox imbalance as a novel hallmark of SMA beyond SMN deficiency. A schematic was created in BioRender. Vrettou, S. (2026) https://BioRender.com/c5hob0j. BioRender publication license: *MV29AWI177.*

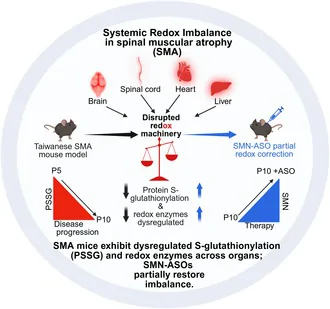

## Abbreviations

**ASO**, Antisense oligonucleotides

**CNS**, Central nervous system

**DTT**, Dithiothreitol

**GPX4**, Glutathione peroxidase 4

**GRX1,2**, Glutaredoxin 1 and 2

**GSH**, glutathione

**GSR**, Glutathione reductase

**GST**, Glutathione‐S‐transferase

**HET**, Heterozygous

**NEM**, N‐ethylmaleimide

**P1**, postnatal day 1

**P10**, postnatal day 10

**P5**, postnatal day 5

**PSSG**, Protein S‐glutathionylation

**ROS**, Reactive oxygen species

**SMA**, Spinal muscular atrophy

**SMN**, Survival motor neuron protein

**SMN1,2**, Survival motor neuron genes 1 and 2

**WT**, Wild‐type

Spinal muscular atrophy (SMA) is a severe neuromuscular disorder caused by homozygous deletion or mutation of the *SMN1* gene, resulting in reduced levels of survival motor neuron (SMN) protein [1]. All patients with SMA lack fully functional *SMN1* alleles, either by homozygous deletion or by loss‐of‐function mutation, and rely on *SMN2*, a nearly identical paralog gene that, due to a silent mutation in exon 7, predominantly produces transcripts lacking exon 7 [2, 3]. The number of *SMN2* copies is a strong modifier of the disease severity, with higher copy number correlating with milder phenotypes [2]. Recently, it has been emphasized that SMA should be viewed as a disease spectrum shaped not only by *SMN2* copies but also by additional genetic modifiers and the timing and nature of therapeutic intervention [2].

Several disease‐modifying therapies that enhance SMN production have reached the patients, including antisense oligonucleotides, small molecules, and gene therapy. While these interventions have dramatically altered SMA disease progression, a subset of patients remain partial or nonresponders, and residual disease manifestations persist even in treated individuals [4, 5, 6, 7]. This highlights the urgent need to uncover complementary, SMN‐independent mechanisms that contribute to pathology.

The SMN protein forms part of a large macromolecular complex required for spliceosomal small nuclear ribonucleoprotein (snRNP) assembly and pre‐mRNA splicing, ensuring correct RNA processing in all cell types [8, 9]. Mechanistically, SMN protein plays fundamental roles beyond snRNP biogenesis, including regulation of mRNA transport and translation, axonal local translation, cytoskeletal dynamics, and mitochondrial trafficking and maintenance [10, 11, 12, 13, 14, 15, 16]. Loss of SMN disrupts axonal mRNA transport and local translation, leading to reduced mitochondrial density, impaired respiration, and accumulation of reactive oxygen species [10, 17, 18, 19]. These mitochondrial and metabolic alterations provide a direct mechanistic link between SMN deficiency and redox disequilibrium. Understanding how restoration of SMN expression influences these processes is therefore essential to interpret systemic oxidative‐stress signatures and to define the extent to which they are modulated under therapeutic intervention.

Beyond the neuromuscular system and in line with the diverse role of the ubiquitously abundant SMN protein, SMA manifests as a systemic disorder in SMA mice and humans [20, 21]. Increasing evidence demonstrates that oxidative stress and mitochondrial dysfunction are recurrent features, with impaired respiratory chain activity, reduced ATP production, and elevated reactive oxygen species (ROS) detected in both neurons and peripheral tissues [22, 23]. Although these aspects are not the main focus of this study, they underscore the link between redox imbalance and SMA pathogenesis.

More broadly, SMA shares key hallmarks with other neurodegenerative disorders in which redox signaling cascades play a central role, including amyotrophic lateral sclerosis, Parkinson's disease, and Alzheimer's disease. As summarized in our recent review, excessive ROS, dysregulated thiol chemistry, and impaired antioxidant defenses represent converging mechanisms across neurodegeneration [24]. Yet, despite the centrality of redox regulation, systemic analyses of redox‐sensitive reversible protein modifications in SMA have not been performed.

Recent functional studies highlight the therapeutic potential of targeting oxidative stress. Thelen et al. demonstrated that rebalancing redox and metabolic stress using *N*‐acetylcysteine (NAC) or pyruvate alleviates oxidative stress and increases SMN protein levels in SMA murine motor neurons [25]. Their proteomic data also revealed upregulation of mitochondrial glutaredoxin‐2 (GRX2) in SMA primary neurons, suggesting adaptive changes in the glutathione system. However, these investigations were restricted to cultured motor neurons and did not address systemic tissues.

Protein S‐glutathionylation, the reversible conjugation of glutathione to protein cysteine residues, is a key redox modification that shields thiols from irreversible oxidation and regulates protein function [26]. In several diseases, disruption of enzymes that regulate S‐glutathionylation cycling, such as glutaredoxins or glutathione transferases, phenocopies hallmark pathological features [24, 27]. Here, we apply a comprehensive analysis of S‐glutathionylated proteins for the first time in SMA as a molecular ‘footprint’ of redox imbalance. By studying both early symptomatic and late symptomatic stages, and analyzing neuronal (brain, spinal cord) and peripheral (heart, liver, and skeletal muscle) tissues, we provide a comprehensive view of systemic redox modifications in SMA. Furthermore, we evaluate how mild SMN restoration by SMN‐targeting antisense oligonucleotides (SMN‐ASO) modulates these redox signatures across tissues, thereby linking protein thiol homeostasis to therapeutic response.

## Materials and methods

### Animals

**Fig. 1:**
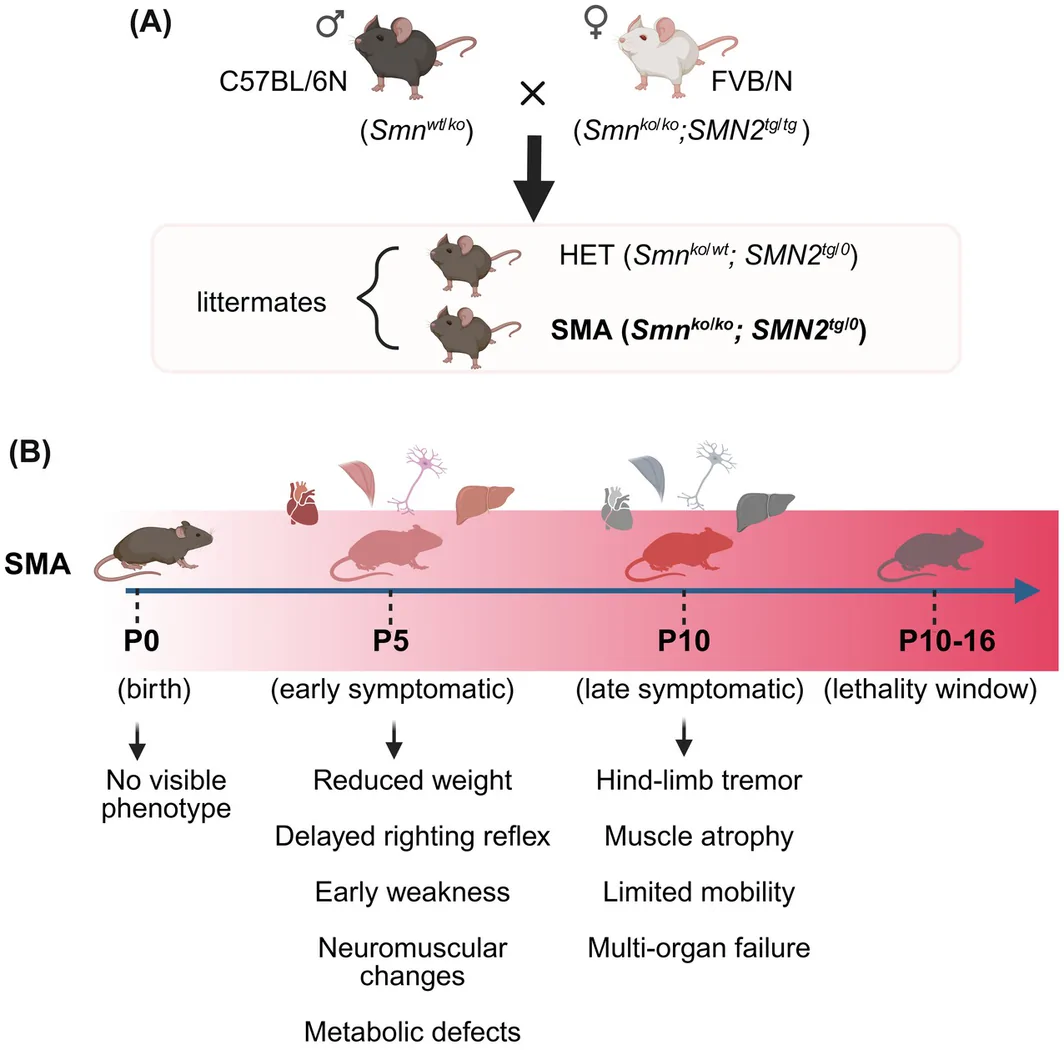
Breeding scheme and phenotypic progression of the Taiwanese SMA mouse model. (A) SMA mice were generated by crossing male C57BL/6N (*Smn* ^wt*/ko*^) with female FVB/N (*Smn* ^ko*/ko*^ *; SMN2* ^*tg/tg*^) carriers. The resulting litters include heterozygous (HET, *Smn* ^wt/ko^, *SMN2* ^tg/0^) and homozygous SMA (*Smn* ^ko/ko^, *SMN2* ^tg/0^) offspring expressing the human *SMN2* transgene. (B) At P0 (postnatal day 0), no overt phenotype is observed. By P5 (postnatal day 5; early symptomatic stage), mice exhibit reduced body weight, delayed righting reflex, early weakness, and emerging neuromuscular alterations. By P10 (postnatal day 10; late symptomatic stage), animals display hind‐limb tremor, pronounced muscle atrophy, limited mobility, and systemic multi‐organ failure, preceding the lethality window (P10–16; postnatal day 10–16). Schematics were created in BioRender. Vrettou, S. (2026) https://BioRender.com/c5hob0j. BioRender publication license: *BO29CDB64S*.

The severe Taiwanese SMA mouse model, originally described by Hsieh‐Li *et al*. [28], was maintained on a C57BL/6N background for more than seven generations to ensure genetic uniformity and bred according to Riessland *et al*. [29]. This SMA model exhibits an early onset phenotype due to homozygous murine *Smn1 knockout* combined with two copies of the human *SMN2* transgene inserted on one allele. Experimental cohorts consisted of wild‐type (WT, *Smn* ^wt/wt^, without the human *SMN2* transgene), heterozygous (HET, *Smn* ^wt/ko^, *SMN2* ^tg/0^), and SMA (*Smn* ^ko/ko^, *SMN2* ^tg/0^) mice as overviewed in Fig. 1A. SMA and HET mice were obtained as littermates, while WT controls were bred separately but organs harvested at matching postnatal days. The breeding scheme used to generate HET and SMA littermates is illustrated in Fig. 1A, providing a visual overview of the cross between C57BL/6N (*Smn* ^wt*/ko*^) and FVB/N (*Smn* ^ko*/ko*^ *; SMN2* ^*tg/tg*^) lines and the resulting genotypes used in this study [30]. All tissues were snap‐frozen on dry ice immediately after dissection and processed in parallel across groups. All mice were housed in the animal facility of the Institute of Genetics, University of Cologne under a 12‐h light/dark cycle with access to food and water *ad libitum*. Breeding, housing, and experimental use of animals were performed in a specific pathogen‐free environment. All animal experiments were approved by the Landesamt für Natur, Umwelt, und Verbraucherschutz Nordrhein‐Westfalen (LANUV) under the application numbers: 81‐02.04. 2020.A196, 81‐02.04. 2019.A017, 81‐02.04. 2019.A138, §4.23.008, §4.22.002.

All animal procedures were designed and conducted in accordance with the ARRIVE 2.0 guidelines and with the principles of replacement, reduction, and refinement (3Rs). Animal numbers were minimized by using littermate‐controlled experimental designs and by harvesting multiple organs from each animal for parallel biochemical analyses. To reduce suffering, mice were monitored daily for general health, body weight, mobility, and neurological symptoms. Animals exhibiting severe tremor, pronounced immobility, failure to thrive, or markedly reduced body weight were not included in experimental analyses and were humanely euthanized according to predefined ethical endpoints. Mice were euthanized rapidly by decapitation at defined neonatal postnatal stages, in accordance with German animal welfare regulations and approved protocols. This method was selected as the most appropriate for neonatal mice to ensure immediate loss of consciousness and to preserve tissue integrity for redox‐sensitive biochemical analyses. All efforts were made to minimize animal distress, and no procedures associated with prolonged suffering were performed.

### Phenotypic staging

In the Taiwanese severe SMA line, the early symptomatic stage (P5, postnatal day 5) corresponds to the onset of mild weight reduction, delayed righting reflex, and early molecular alterations before overt paralysis. The late symptomatic stage (P10, postnatal day 10) represents advanced disease characterized by pronounced muscle weakness, tremor, and loss of motor coordination, with survival typically limited to P10–16 (Fig. 1B). These phenotypic definitions are consistent with prior characterizations of the Taiwanese SMA line [28, 31, 32, 33, 34] and parallel observations in related SMA models documenting early neuromuscular, motor defects, heart, intestine, lung, and skeletal muscle vasculature abnormalities [32, 34]. The same clinical and histopathological criteria were used here to define the experimental time points analyzed in this study as well as the inclusion of non‐neuronal organs such as heart, gastrocnemius, and liver.

### Generation of a milder SMA model

To generate a partial rescued phenotype, a subset of SMA and HET littermates received a single subcutaneous injection of low‐dose SMN‐restoring antisense oligonucleotides (SMN‐ASOs; 30 μg in sterile saline) into the skin fold at the back of the neck at postnatal day 1 (P1), following previously validated protocols [30, 35, 36]. The ASO, targeting the *SMN2* intronic splicing silencer N1 (ISS‐N1), promotes inclusion of exon 7 in *SMN2* pre‐mRNA by blocking repressor binding to the ISS‐N1 site, thereby partially restoring the production of full‐length, functional SMN protein from the *SMN2* gene [37]. This produced SMA + SMN‐ASO and HET + SMN‐ASO groups with mild SMN upregulation, as previously described [38]. This sub‐optimal regimen yields a modest but reproducible increase in SMN, sufficient to assess downstream pathways without fully normalizing the phenotype. This resembles the situation of about half of all SMA patients, who carry only 2 *SMN2* copies and are treated with Nusinersen (SMN‐ASOs) [2]. The ASO group received vehicle (PBS/saline) injections at identical timing, route, and volume. A nontargeting (‘scrambled’) ASO was not used, as such oligonucleotides may produce off‐target hybridization or innate‐immune effects [39, 40] that could confound subtle redox and mitochondrial outcomes in neonatal tissues. Vehicle injections reproduce only handling and injection stress, representing the most appropriate control for this developmental stage. Although survival analysis was not included in this study, previous work in the Taiwanese SMA line has shown that systemic neonatal ASO treatment significantly extends lifespan and alleviates neuromuscular pathology [38, 41]. Low‐dose SMN‐ASO regimens have routinely been used in combinatorial paradigms to generate a mild‐rescue background that partially improves survival and motor function [30, 35, 36].

Genotyping was performed on tail DNA collected at the time of sacrifice (P5 or P10), using these primers: *Smn* ^KO^ forward: 5′‐ATAACACCACCACTCTTACTC‐3′; *Smn* ^KO^ reverse 1: 5′‐AGCCTGAAGAACGAGATCAGC‐3′; *Smn* ^KO^ reverse 2: 5′‐TAGCCGTGATGCCATTGTCA‐3′. All experimental groups contained at least three biological replicates (*n* ≥ 3).

### Tissue collection and homogenization

Brain, spinal cord, heart, liver, and gastrocnemius whole organs were snap‐frozen on dry ice immediately after dissection and stored at −80 °C until use. For protein extraction, tissues were homogenized in ice‐cold RIPA buffer (50 mm Tris–HCl pH 7.4, 150 mm NaCl, 1% NP−40, 0.1% SDS, 0.5% sodium deoxycholate) supplemented with protease and phosphatase inhibitor cocktails (Cat#: A32961, Thermo Fisher Scientific, Waltham, MA, USA) as well as 50 mm *N*‐ethylmaleimide (NEM, Cat#: 23030, Thermo Fisher Scientific). Homogenization was performed in Precellys Peqlab tubes using a Peqlab homogenizer (Bertin Technologies SAS, Montigny‐le‐Bretonneux, France). Homogenates were kept on ice for 10 min and centrifuged at maximum speed (~18 000 ***g***) for 20 min at 4 °C. Supernatants were then transferred to 1.5 mL Eppendorf tubes, sonicated at 4 °C (medium intensity, for 7 min), and centrifuged again at maximum speed (~18 000 ***g***) for 20 min at 4 °C. The new supernatants from the last step were transferred again to new 1.5‐mL Eppendorf tubes and protein concentrations were determined by Bradford assay.

### Western blotting reducing conditions (enzyme expression)

For detection of redox enzymes, 30–40 μg of protein was mixed with 1x Laemmli buffer containing 5% β‐mercaptoethanol, heated at 95 °C for ≤ 3 min, and resolved on homemade 12% or 4–20% SDS/PAGE gradient gels (Cat#: 4561094, Bio‐Rad Laboratories, Inc., Hercules, CA, USA). Proteins were transferred to PVDF or nitrocellulose membranes using the Bio‐Rad Trans‐Blot® Turbo™ Transfer System, blocked with 5% (w/v) non‐fat milk in TBST (20 mm Tris–HCl pH 7.6, 150 mm NaCl, 0.1% Tween‐20) for ≥ 1 h at room temperature. Primary antibodies were incubated either for 2 h at room temperature or overnight at 4 °C in TBST plus 5% milk. After washing (3 × 5 min, in TBST), membranes were incubated with HRP‐conjugated secondary rabbit (1 : 3000, Cat#: 7074, Cell Signaling Technology, Inc., Danvers, MA, USA) or mouse (1 : 5000, Cat#: 115‐035‐003, Dianova, Jackson ImmunoResearch Laboratories, Inc., West Grove, PA, USA) antibodies for at least 1 h at room temperature and washed again (3 × 5 min).

Proteins were visualized using the Thermo Scientific™ SuperSignal™ West Pico PLUS chemiluminescent substrate (Cat#: 34580). Primary antibody targets included glutathione reductase (GSR, mouse, monoclonal, Cat#: 133245, Santa Cruz Biotechnology, Inc., Dallas, TX, USA), glutaredoxin‐1 (GRX1, rabbit, polyclonal, Cat#: 45953, Abcam plc, Cambridge, UK), glutaredoxin‐2 (GRX2, rabbit, polyclonal, Cat#: 191292, Abcam), glutathione‐S‐transferase p (GSTp, rabbit, polyclonal, Cat#: 312, MBL, Medical & Biological Laboratories Co., Ltd., Nagoya, Japan), glutathione peroxidase‐4 (GPX4, rabbit, monoclonal, Cat#: 125066, Abcam), survival motor neuron protein (SMN, mouse, monoclonal, Cat#: 610647, BD Biosciences Transduction Laboratories™). The SMN antibody recognizes an epitope located within the N‐terminal region of the human SMN protein, corresponding to the portion encoded by exon 1 or 2 [42]. Only full‐length SMN protein is stable while Δ7SMN is unstable and rapidly degraded [43]. Vinculin (rabbit, monoclonal, Cat#: 129002, Abcam) was used as loading control. All primary antibodies were used in a 1 : 1000 dilution in 5% non‐fat milk in TBST.

### Nonreducing conditions (*in vivo* detection of S‐glutathionylated proteins)

To detect protein S‐glutathionylation (PSSG), 30–40 μg of protein was mixed with 1× Laemmli buffer without reducing agents and not heated before loading. Separation was carried out under nonreducing SDS/PAGE conditions, followed by immunoblotting with antiglutathione (GSH, mouse, Cat#: ab19534, Abcam) antibody (1 : 1000 dilution), as described above. Loading control normalization was performed using the Thermo Fisher Scientific Reversible Protein Stain Kit for PVDF Membranes (Cat#: 24585). Proteins were visualized by chemiluminescence, as described above.

### Lipid peroxidation assay (TBARS)

Lipid peroxidation was quantified as thiobarbituric acid reactive substances (TBARS) using the colorimetric method of the Lipid Peroxidation (MDA) Assay kit (Cat#: ab118970, Abcam) based on the operating manual. Malondialdehyde (MDA) standards were used for calibration, and values were expressed as MDA equivalents (nanomoles) normalized to protein content (milligram of protein). For P10 analyses, brain, spinal cord, and heart whole homogenates from WT, HET, and SMA mice were assayed (three biological replicates per group). In addition, heart tissues from HET, SMA, HET + ASO, and SMA + ASO groups were analyzed in five independent TBARS experiments (*n* ≥ 4 biological replicates) to assess the effect of SMN‐ASO treatment. All samples were processed in parallel, and protein concentration was determined using the Bradford assay.

### Statistics

All data are represented as mean ± SD from ≥ 3 biological replicates. Statistical analyses were performed using GraphPad Prism version 10.6.1 (GraphPad software). Comparisons between two groups were performed using an unpaired two‐tailed Student's *t*‐test, applied specifically to assess SMA vs. SMA + ASO for SMN rescue within each organ. For comparisons involving three groups (WT, HET, SMA), one‐way ANOVA with Tukey's post hoc test was applied. Datasets including ASO‐treated groups (WB for HET, SMA, HET + ASO, SMA + ASO) were analyzed using two‐way ANOVA with genotype and treatment as factors, followed by Tukey's post hoc test. Statistical significance was denoted as follows: *P* < 0.05 (*), *P* < 0.01 (**), *P* < 0.001 (***), *P* < 0.0001 (****).

## Results

### Validation of the detection strategy for protein S‐glutathionylation

Protein S‐glutathionylation represents the reversible conjugation of glutathione to protein thiols, serving both as a redox buffer and as a regulatory post‐translational modification [26]. By transiently protecting cysteine residues from irreversible oxidation and modulating protein activity, PSSG acts as a sensitive molecular indicator of thiol‐redox equilibrium and oxidative stress.

**Fig. 2:**
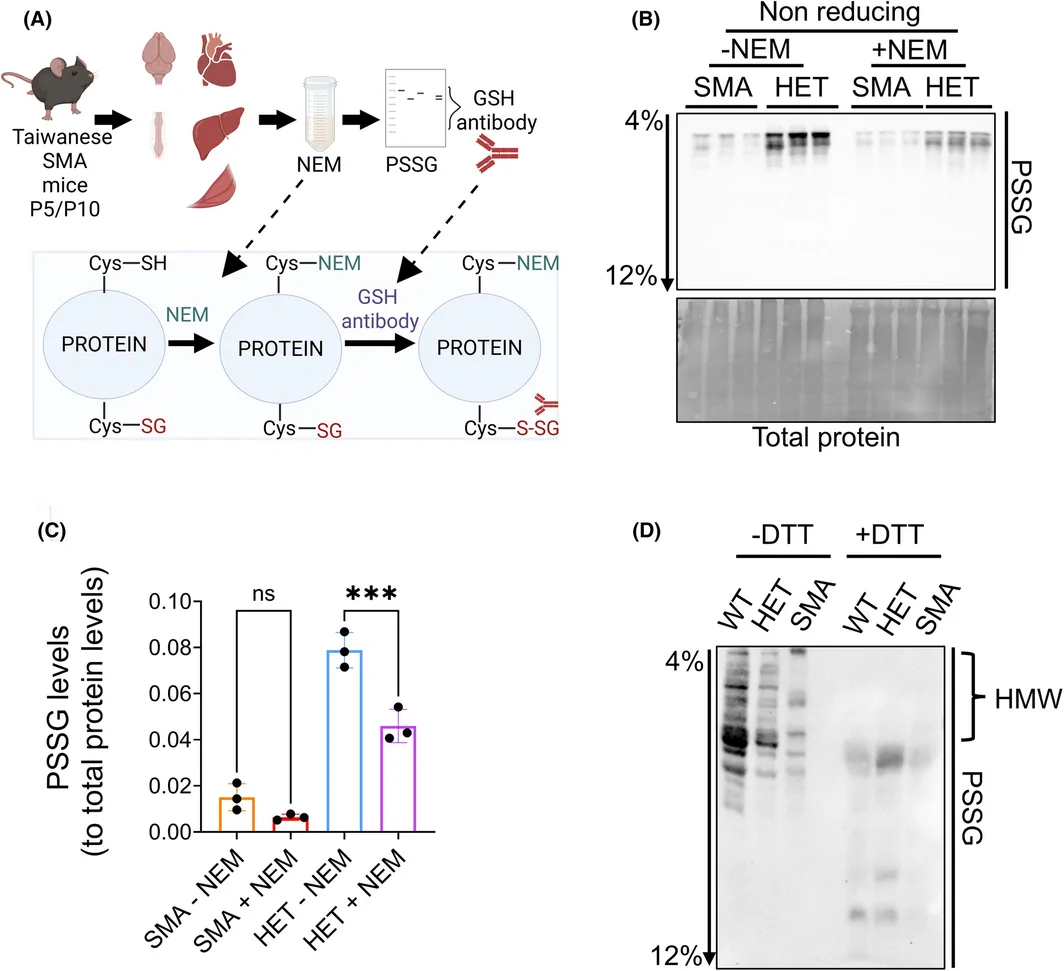
Establishing the protocol for *in vivo* detection of the S‐glutathionylated proteins in the Taiwanese SMA mouse model. (A) Schematic representation of the experimental workflow created in BioRender. Vrettou, S. (2026) https://BioRender.com/c5hob0j. BioRender publication license: *UJ29AWH29V*. Organs (brain, spinal cord, heart, liver, gastrocnemius muscle) were collected from Taiwanese SMA mice and littermate controls at postnatal day 5 (P5) or 10 (P10). Tissue lysates were homogenized in buffer containing 50 mm N‐ethylmaleimide (NEM) to alkylate free thiols and prevent post‐lysis thiol–disulfide exchange. Proteins were separated under non‐reducing SDS–PAGE and probed with an antiprotein S‐glutathionylation (PSSG) antibody. (B) Representative immunoblots of skeletal muscle lysates from SMA and heterozygous (HET) animals processed with or without NEM. A total protein reversible staining is shown as a loading control. (C) Quantification of total PSSG signal normalized to total protein content. Expression of PSSG + NEM relative to the –NEM condition within each genotype is shown (*n* = 3 biological replicates per group; mean ± SD; ***P < 0.001; ns, not significant). (D) Antibody specificity validation using SMA, HET, and WT brain lysates under reducing (+100 mm DTT, +50 mm NEM) versus non‐reducing (–DTT, +50 mm NEM) conditions. Under reducing conditions, PSSG bands were abolished, confirming recognition of disulfide‐linked glutathionylated proteins. Gels were run at 4–12% gradient to resolve both high‐molecular‐weight (HMW) aggregates and lower‐molecular‐weight proteins.

To initiate an organ‐specific characterization of the total S‐glutathionylated proteome, we used the Taiwanese SMA mouse model and harvested multiple neuronal (brain, spinal cord) and non‐neuronal (heart, liver, gastrocnemius) organs at P5, an early symptomatic stage, and P10, a late symptomatic stage (Fig. 2A). Tissues were homogenized in RIPA buffer supplemented with 50 mm NEM, an alkylating agent that preserves thiol modifications [44], and protein lysates were resolved under non‐reducing conditions and probed with anti‐GSH antibody to detect the *in vivo* glutathionylated proteins (PSSG) (Fig. 2A,B). We first confirmed the importance of NEM inclusion, which minimized significantly overoxidation artifacts, especially at the HET control group, and stabilized PSSG (Fig. 2B,C). In addition, treatment with 100 mm DTT abolished the high‐molecular‐weight (HMW) disulfide‐linked signals, confirming antibody specificity (Fig. 2D). These controls established a robust workflow, and all subsequent experiments for PSSG detection were performed on NEM‐homogenized lysates under nonreducing conditions, while analyses of other protein targets were carried out under standard reducing conditions.

### Early symptomatic stage (P5) reveals reduced protein S‐glutathionylation levels in specific SMA organs

**Fig. 3:**
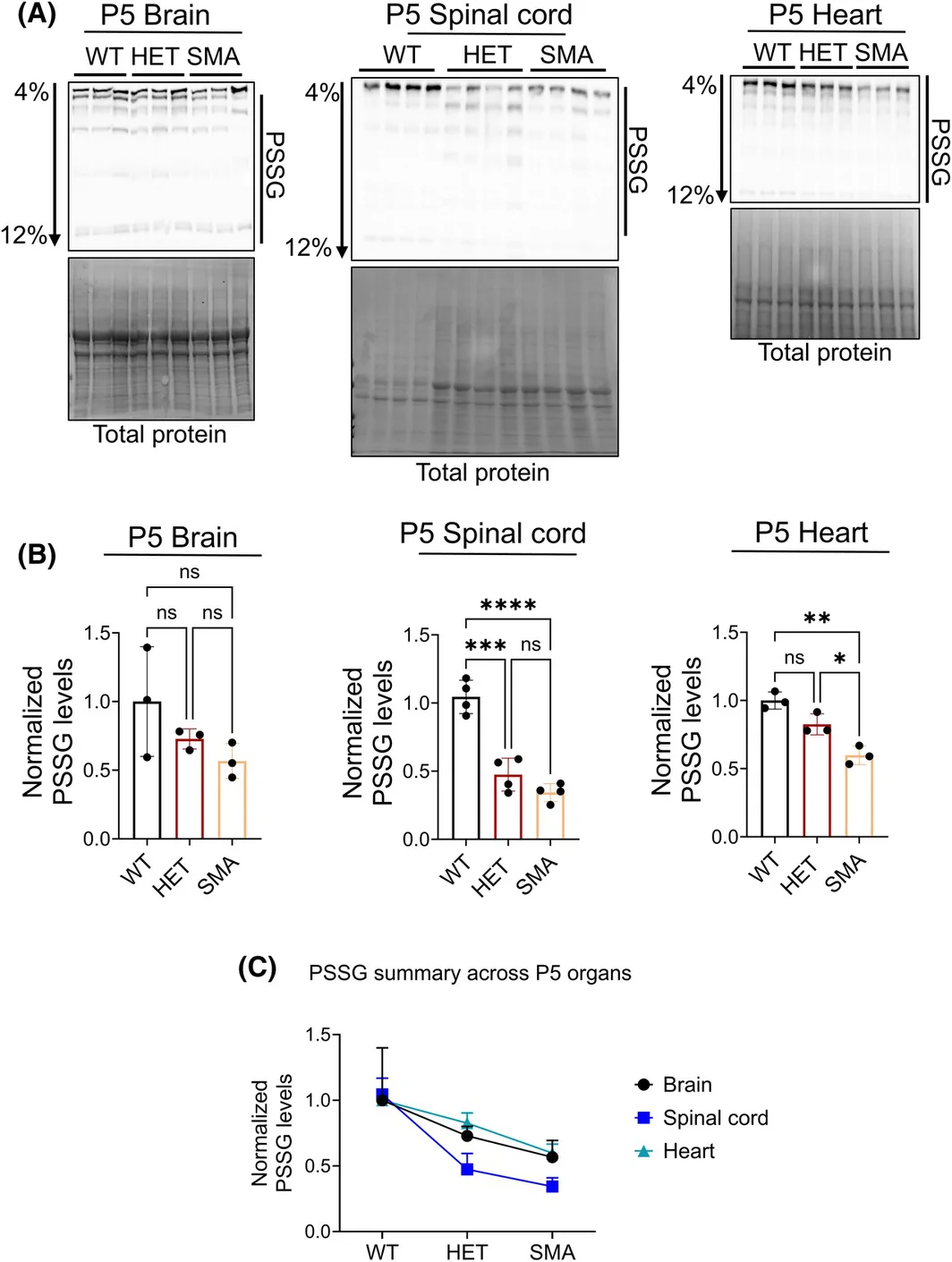
S‐glutathionylation levels in multiple organs at early symptomatic stage (P5) of SMA mice. (A) Representative non‐reducing western blots showing protein S‐glutathionylation (PSSG) in brain, spinal cord, and heart lysates from wild‐type (WT), heterozygous (HET), and SMA littermates at P5. Total protein loading is shown by total protein reverse staining. (B) Quantification of normalized PSSG signal (to total protein) across genotypes for each organ. Spinal cord and heart of SMA mice showed significant reductions in PSSG compared to WT, whereas the brain did not exhibit significant changes (*n* = 3–4 biological replicates per group; mean ± SD; *P < 0.05, **P < 0.01, ***P < 0.001, ****P < 0.0001; ns, not significant). (C) Summary slope graph illustrating the overall trend of PSSG levels across brain, spinal cord, and heart at P5. Symbols represent mean values for each organ and genotype, and error bars indicate the standard deviation derived from individual western blot measurements.

Having validated the specificity and stability of the S‐glutathionylated proteins' detection approach, we next examined PSSG in different organs collected at P5 to identify early alterations in thiol‐redox homeostasis in the SMA model (Fig. 3A). Quantification of nonreducing western blots showed that PSSG levels were significantly reduced in SMA compared to WT in the spinal cord, heart (Fig. 3B) and liver (Fig. S1A), and in gastrocnemius of SMA compared to HET mice (Fig. S1B). In the spinal cord, both HET and SMA were significantly lower than WT, with SMA not differing from HET (Fig. 3B). In the heart, SMA mice displayed a reduction relative to both WT and HET controls, whereas WT and HET were comparable (Fig. 3B). No significant changes were observed in the brain (Fig. 3B). A slopegraph summary emphasized the overall downward trend from WT to HET to SMA, with spinal cord and heart showing the most pronounced early decreases (Fig. 3C). Liver also displayed a consistent reduction, while gastrocnemius presented a drop from HET to SMA (Figs S1 and S2).

### Late symptomatic stage (P10) harbors further reduction of protein S‐glutathionylation across central and peripheral SMA tissues

**Fig. 4:**
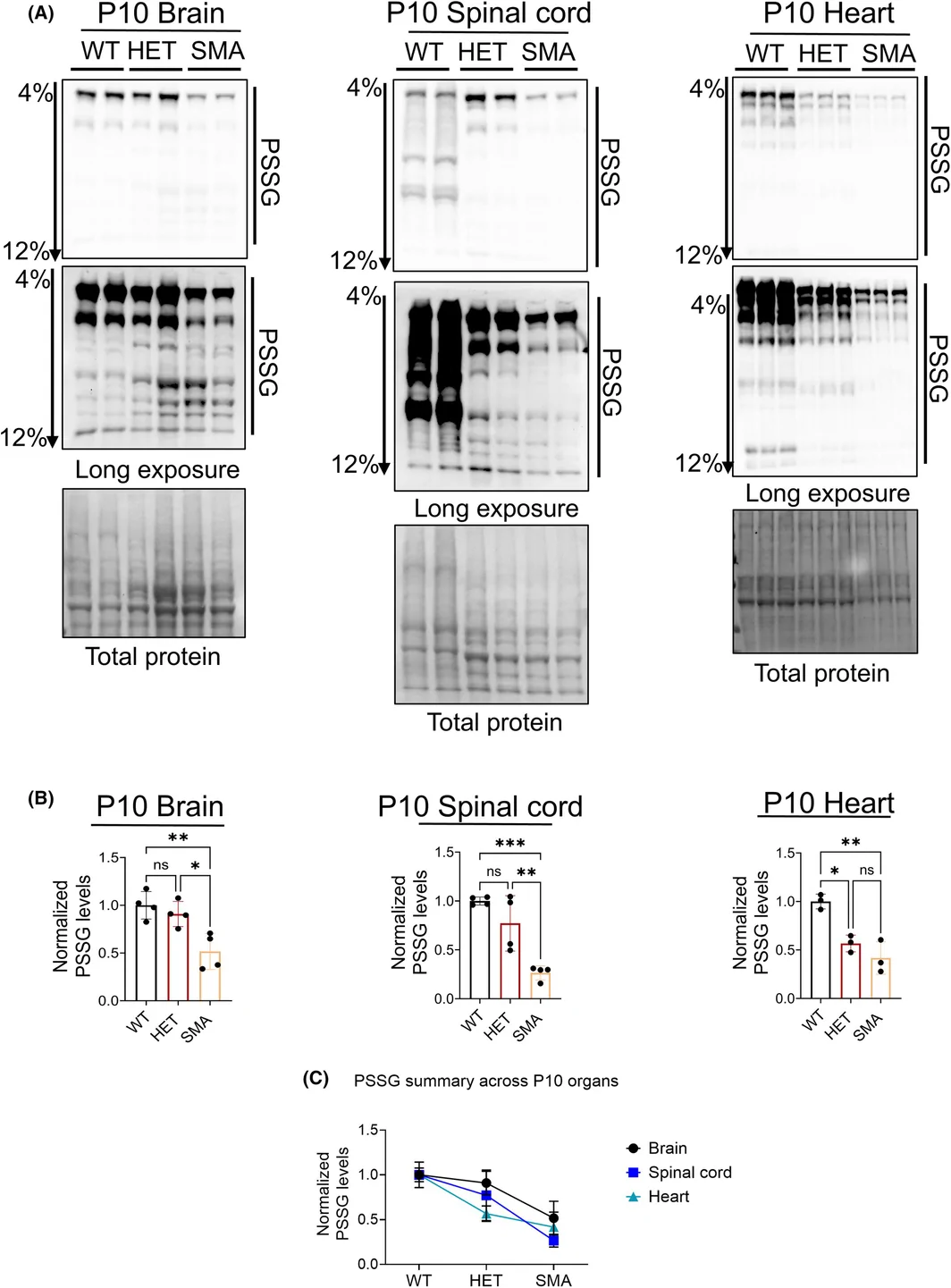
S‐glutathionylation levels in multiple organs at late‐symptomatic stage (P10) of SMA mice. (A) Representative non‐reducing western blots (low and long exposure) showing protein S‐glutathionylation (PSSG) in brain, spinal cord, and heart lysates from wild‐type (WT), heterozygous (HET), and SMA littermates at P10. Total protein loading is shown by total protein reverse staining. (B) Quantification of normalized PSSG signal (to total protein) across genotypes for each organ. SMA brain, spinal cord, and heart displayed significant decreases compared to WT and/or HET (*n* = 3–4 biological replicates per group; mean ± SD; *P < 0.05, **P < 0.01, ***P < 0.001; ns, not significant). (C) Summary slope graph illustrating the overall trend of PSSG levels across brain, spinal cord, and heart at P10. Symbols represent mean values for each organ and genotype, and error bars indicate the standard deviation derived from individual western blot measurements.

To follow the status of protein S‐glutathionylation at the late symptomatic stage, we next analyzed PSSG levels at P10 (Fig. 4A,B, Fig. S3A,B). Significant reductions were observed in the brain, spinal cord, heart (Fig. 4B), and liver (Fig. S3A), but not gastrocnemius (Fig. S3B). In the brain, SMA displayed lower levels of PSSG than both WT and HET, with HET and WT remaining similar (Fig.4B). In the spinal cord, SMA PSSG levels were reduced compared to WT and HET, whereas HET PSSG levels were not significantly different from WT (Fig.4B). In the heart, SMA PSSG levels were significantly lower than WT, with HET changes not significant compared to SMA but significant compared to WT (Fig. 4B). The liver PSSG levels exhibited the same pattern, with SMA showing reduction compared to WT but not differentiating from HET (Fig. S3A), but gastrocnemius showed no changes in between the groups (Fig. S3B).

These findings indicate that decreases in protein S‐glutathionylation emerge early in the SMA spinal cord, heart, and liver, and worsen as disease progresses. In contrast, in brain, a profound decrease in protein S‐glutathionylation was only found at the late symptomatic stage (P10). Gastrocnemius muscle data showed no apparent effect at the late symptomatic stage. These observations are presented at the slopegraph summaries (Fig. 4C and Fig. S4).

### Redox enzyme remodeling and GPX4 loss reveal a ferroptotic signature in SMA heart

**Fig. 5:**
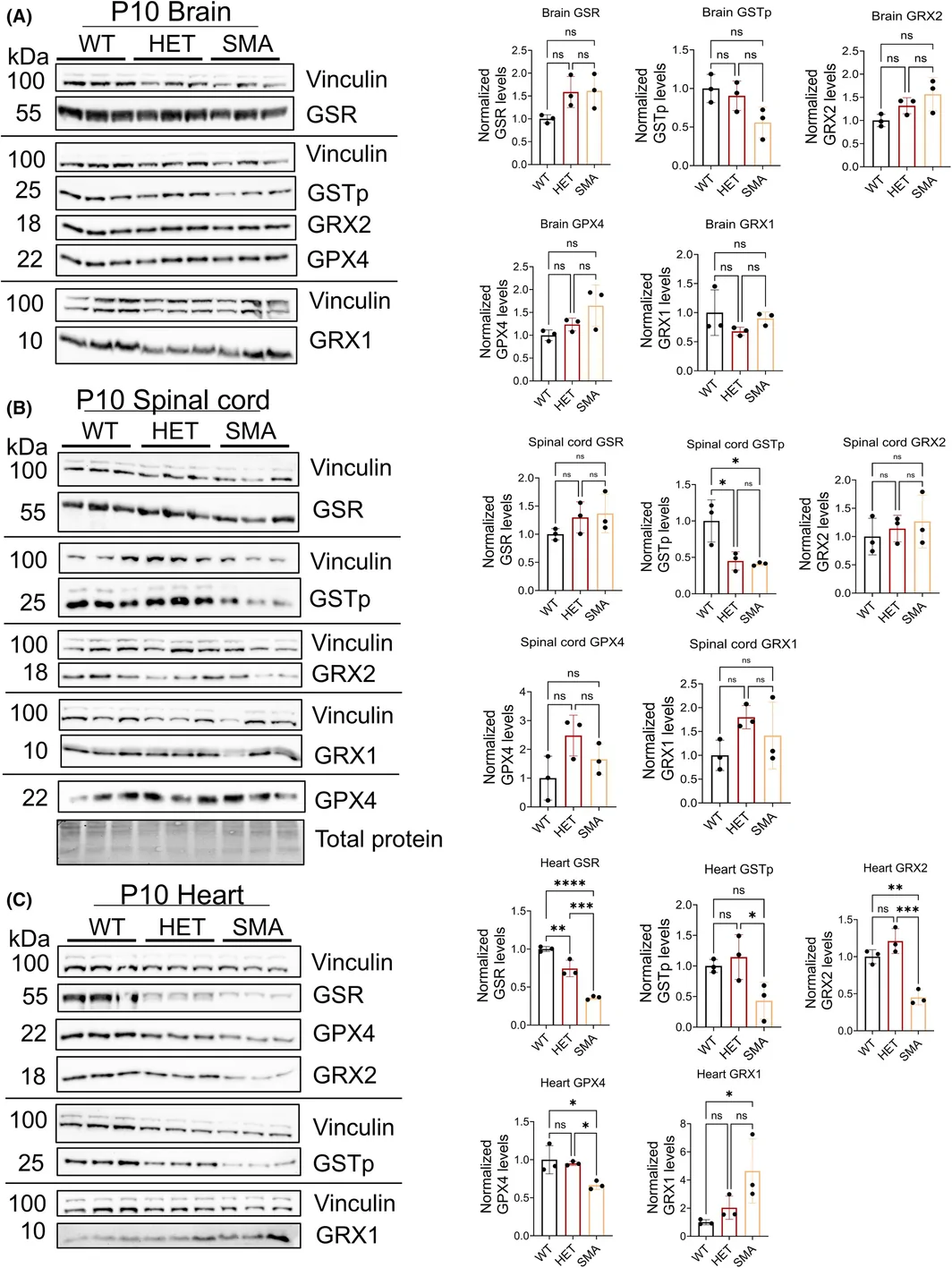
Organ‐specific remodeling of redox enzymes at the symptomatic stage (P10) in SMA mice. (A–C) Representative western blots and corresponding heatmaps for components of the glutathione redox machinery in brain (A), spinal cord (B), and heart (C) from wild‐type (WT), heterozygous (HET), and SMA mice at P10. Enzymes analyzed: glutathione reductase (GSR), glutathione S‐transferase p (GSTp), glutaredoxin‐1 (GRX1, cytosolic) and glutaredoxin‐2 (GRX2, mitochondrial), and glutathione peroxidase‐4 (GPX4). Protein signals were normalized to vinculin (or total protein reverse staining) and expressed relative to the WT mean. Molecular weights are indicated in kilodaltons (kDa). (A) In the brain, GSR GPX4 GSTp, GRX1 and GRX2 were unchanged. (B) In the spinal cord, GSTp significantly decreased in SMA relative to both HET and WT, while GSR, GRX1, GRX2, and GPX4 were not different. (C) In the heart, multiple enzymes were altered: GSR, GSTp, GRX2 and GPX4 were significantly decreased, whereas GRX1 significantly increased in SMA compared to WT. GSTp and GPX4 also significantly decreased in SMA compared to HET. Protein expression data were analyzed by one‐way ANOVA followed by Tukey's *post hoc* tests for pairwise comparisons (WT vs HET, WT vs SMA, HET vs SMA). Data are shown as mean ± SD (*n* = 3–4 biological replicates per group; *P < 0.05, **P < 0.01, ***P < 0.001, ****P < 0.0001; ns, not significant).

Following the quantification of protein S‐glutathionylation, we next examined the expression of key redox enzymes that regulate this post‐translational modification within the glutathione system (Fig. 5A). These included glutaredoxins (GRX1, GRX2), which catalyze deglutathionylation reactions; glutathione‐S‐transferase p (GSTp), which facilitates protein S‐glutathionylation of cysteine residues; and glutathione reductase (GSR), a core enzyme that regenerates reduced glutathione and thereby reflects the overall cellular redox capacity [45].

To determine whether enzyme‐level changes precede the symptomatic stage, we first profiled components of the glutathione machinery at P5 across the brain (GSR, GSTp, GRX1, GRX2), spinal cord (GSR, GSTp), and heart (GSR, GRX2) (Figs S5 and S6). At this early symptomatic stage, the brain exhibited decreased GRX1 levels (Fig. S5), while in the heart, GRX2 was significantly reduced in SMA compared to HET (Fig. S6B). All other enzymes remained unchanged, and overall effect sizes were small (Fig. S6). Thus, early decreases in protein S‐glutathionylation occurred alongside subtle, organ‐specific perturbations of the enzymatic network at P5.

At P10, western blot quantification revealed pronounced organ‐specific remodeling (Fig. 5). In the brain, GSR, GRX1, GRX2, and GSTp remained unchanged (Fig. 5A). In the spinal cord, GSTp expression was significantly reduced in SMA relative to controls, while GSR was unchanged (Fig. 5B). In the heart, both GSR and GSTp decreased, GRX1 was elevated, and GRX2 remained reduced in SMA compared with WT and HET (Fig. 5C).

Together, these data demonstrate that SMA at P10 is associated with distinct, tissue‐specific remodeling of the glutathione enzyme network.

Given the defects in core redox enzymes, most evidently in the heart, we next evaluated glutathione peroxidase‐4 (GPX4), a glutathione‐dependent antioxidant enzyme and a canonical ferroptosis marker that links glutathione metabolism to lipid peroxidation [46]. GPX4 expression was markedly decreased in SMA hearts, while remaining unchanged in the brain and spinal cord (Fig. 5 A–C, Figs S7–S9). Consistent with this, TBARS measurements revealed pronounced lipid peroxidation exclusively in the heart, whereas the brain and spinal cord showed negligible or undetectable levels (Fig. S7).

To verify whether other peripheral tissues exhibit similar trends, we also assessed selective enzymes in liver and gastrocnemius (Fig. S8). In the liver, GSR remained unchanged but GSTp and GRX2 levels were increased in SMA relative to controls (Fig. S8A). In the gastrocnemius, both GSTp and GRX2 were unaltered (Fig. S8B), consistent with the absence of significant changes in protein S‐glutathionylation levels shown in Fig. S3B.

Altogether, these findings indicate that SMA at P10 is characterized by organ‐specific remodeling of the glutathione enzyme network, accompanied by GPX4 depletion and lipid peroxidation in the heart, establishing a ferroptotic signature that links glutathione imbalance with impaired antioxidant defense. Notably, the early reduction in mitochondrial GRX2 in the heart at P5 persists in the symptomatic stage, while the initial decrease of cytosolic GRX1 in the brain is abolished by P10, suggesting a progressive, tissue‐dependent adaptation of the redox machinery that parallels disease progression and tissue vulnerability.

### SMN‐ASO treatment partially rescues SMN levels but fails to fully restore protein S‐glutathionylation in SMA organs

**Fig. 6:**
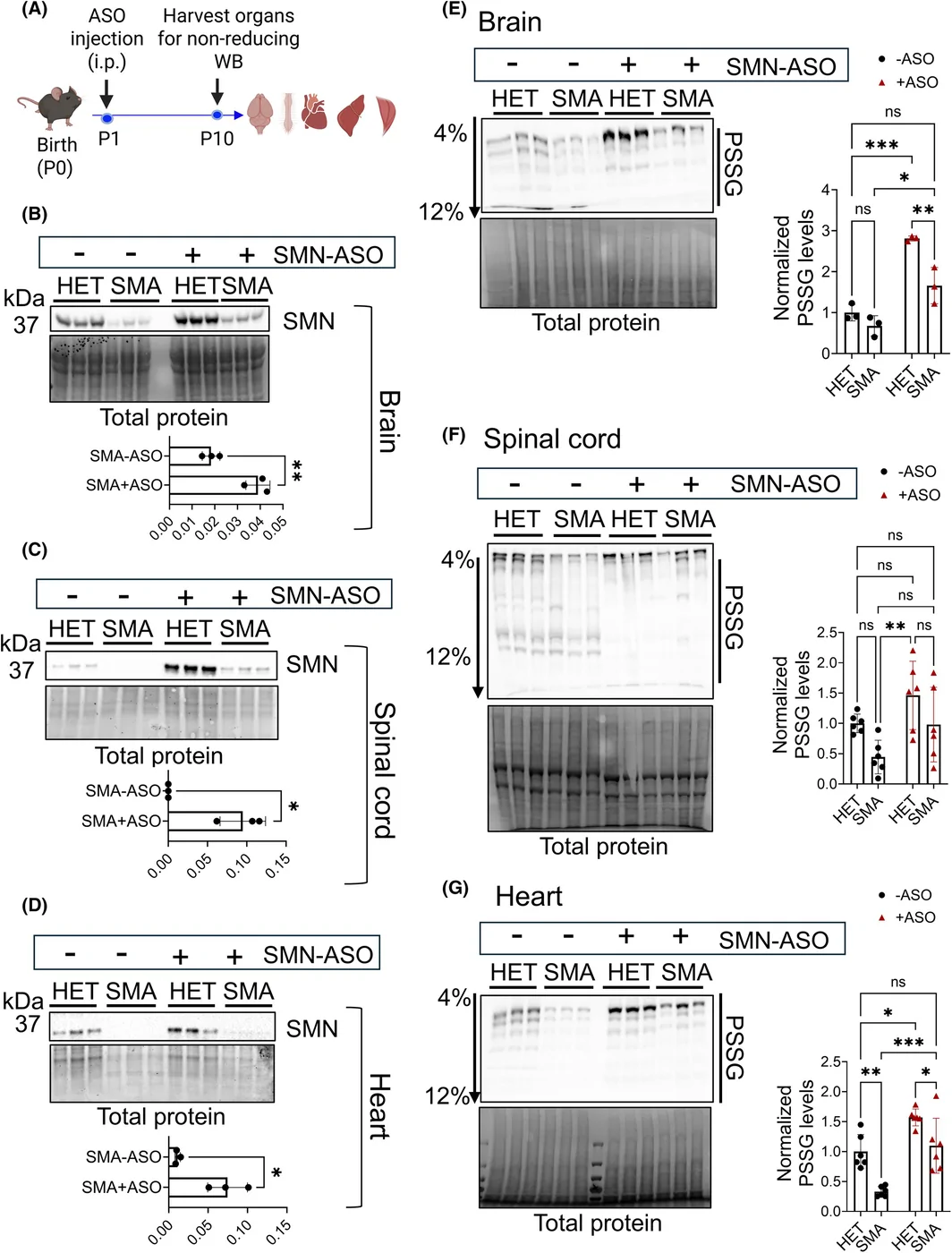
SMN‐ASO treatment partially rescues SMN levels but only modestly restores protein S‐glutathionylation. (A) Experimental design created in BioRender. Vrettou, S. (2026) https://BioRender.com/c5hob0j. BioRender publication license: *PA29AWHH98*. SMA mice received a single antisense oligonucleotide (SMN‐ASO) injection at P1, and organs (brain, spinal cord, heart, liver, gastrocnemius) were harvested at P10 for non‐reducing (PSSG levels) or reducing western blot (WB) analysis. (B–D) Representative WBs showing SMN protein expression in brain, spinal cord, and heart across HET, SMA, SMA + ASO, and HET + ASO groups, with vinculin or total protein as loading control. Corresponding graphs display SMN levels normalized to loading control and compared between SMA and SMA + ASO groups by unpaired *t*‐test. SMN was significantly increased in SMA + ASO brain, spinal cord, and heart. The full two‐way ANOVA including all four genotypes per organ is presented in Fig. S9. Data represents SD (*n* = 3–4 per group). Molecular weights are indicated in kilodaltons (kDa). (E–G) Protein S‐glutathionylation (PSSG) in brain, spinal cord, and heart lysates analyzed by non‐reducing WB using anti‐GSH antibody. Total protein staining is shown below each blot. Quantification revealed increased PSSG levels in SMA + ASO brain and heart compared with SMA corresponding groups, whereas SMA spinal cord PSSG remained unchanged. Statistical analyses were performed by two‐way ANOVA (factors: genotype and treatment). Data are presented as mean ± SD (*n* = 3 biological replicates per group; *P < 0.05, **P < 0.01, ***P < 0.001; ns, not significant).

To assess whether SMN‐ASO therapy modulates the glutathione‐dependent redox balance, SMA and HET littermates were systemically injected with 30 μg SMN antisense oligonucleotides at P1, and organs were collected at P10 for reducing and non‐reducing western blot analyses (Fig. 6A).

As expected, SMN‐ASO treatment increased SMN protein levels across the brain, spinal cord, and heart compared to untreated SMA, confirming efficient yet partial restoration of SMN deficiency (Fig. 6B–D). Two‐way ANOVA followed by Tukey's post hoc test revealed a significant increase of SMN in SMA + ASO compared with untreated SMA in all three organs (*P* < 0.05–0.001) (Fig. S9A–C). HET + ASO animals also exhibited elevated SMN levels relative to untreated HET mice, particularly in the heart, while no significant difference was observed between SMA and SMA + ASO in some peripheral tissues (Fig. S9A–C). In addition, liver and gastrocnemius blots (Fig. S10) showed that SMN‐ASO therapy elevated SMN expression in a modest way in which SMA + ASO values remained statistically indistinguishable from SMA, indicating only partial peripheral rescue.

We next examined whether restoring SMN levels alleviates the redox imbalance by analyzing protein S‐glutathionylation (PSSG) in the same tissues using nonreducing SDS/PAGE (Fig. 6E–G). In the brain, SMA + ASO mice displayed a significant increase in PSSG relative to untreated SMA, although levels remained lower than in HET controls (Fig. 6E). In the spinal cord, PSSG levels were unchanged by ASO treatment, remaining similar between SMA and SMA + ASO groups (Fig. 6B). In the heart, SMN‐ASO treatment produced a significant rise in PSSG compared with SMA, and values reached those of HET (Fig. 6C). Complementary analyses in liver and gastrocnemius (Fig. S11) revealed no significant differences between SMA and SMA + ASO, while the ASO treatment rendered the differences between HET + ASO and SMA + ASO nonsignificant in both liver and gastrocnemius.

Altogether, these results indicate that SMN‐ASO therapy increased SMN levels only mildly and at different magnitudes across the tested organs, leading to partial correction of the glutathione‐dependent redox imbalance. Improvement in S‐glutathionylation was most evident in the heart, liver, brain, and gastrocnemius, whereas the spinal cord showed minimal response.

### SMN‐ASO selectively reduces lipid peroxidation in the heart without fully restoring the redox enzyme network

**Fig. 7:**
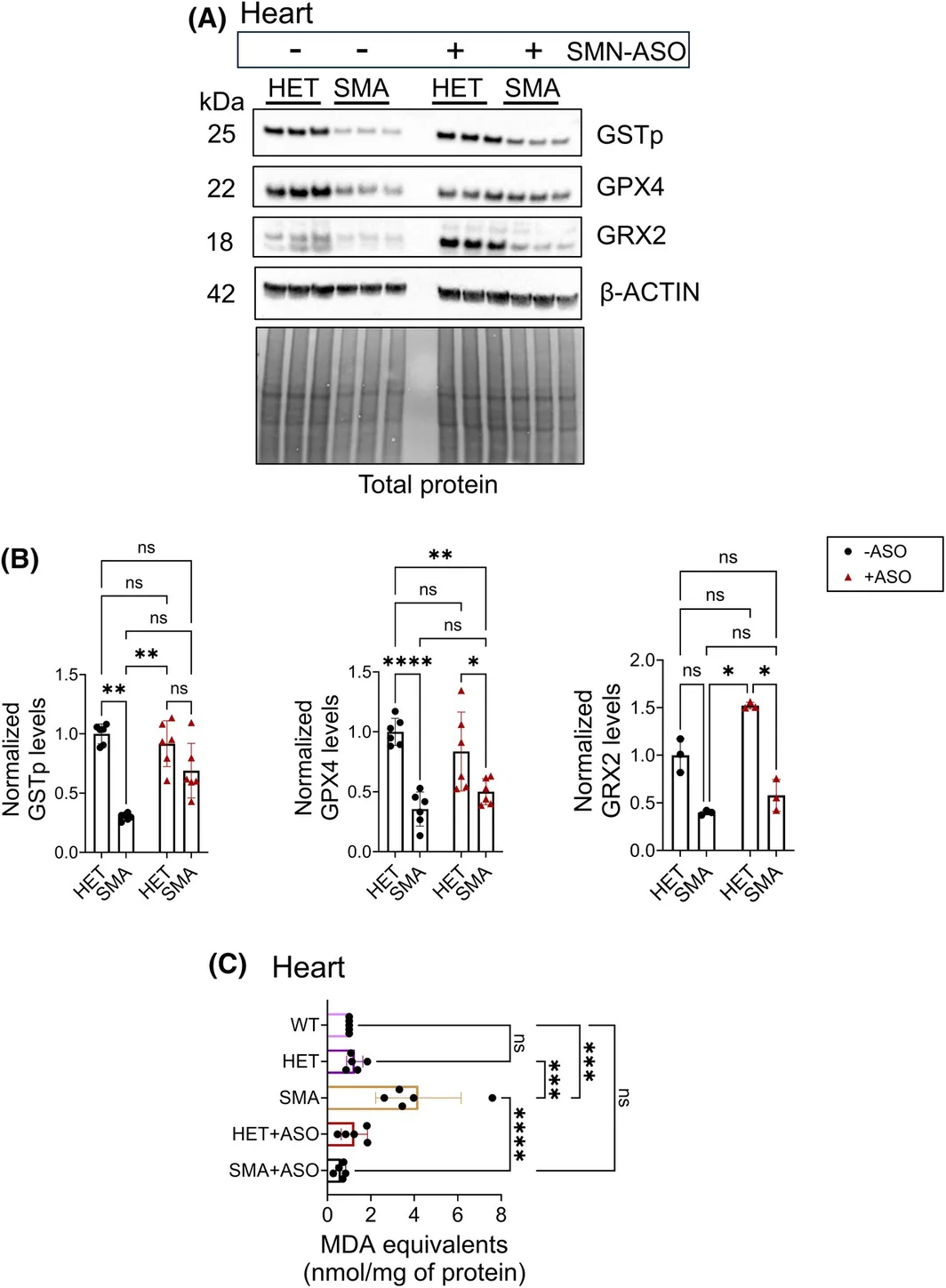
SMN‐ASO treatment selectively reduces lipid peroxidation without fully rescuing the redox enzyme network in SMA. (A, B) Representative western blots and quantification for mitochondrial glutaredoxin (GRX2), glutathione‐S‐transferase‐p (GSTp), and glutathione peroxidase‐4 (GPX4) in heart lysates from HET, SMA, HET + ASO and SMA + ASO at P10. β‐Actin or total protein reverse staining served as loading controls. Protein signals were normalized to loading control and expressed relative to the HET mean. Molecular weights are indicated in kilodaltons (kDa). SMA hearts showed altered GSTp and GPX4 levels compared to HET. SMN‐ASO produced a modest shift in GSTp in SMA, narrowing the difference from HET + ASO, whereas GRX2 remained low and unchanged in SMA + ASO. GPX4 remained reduced in both SMA and SMA + ASO compared with HET. (C) Lipid peroxidation measured by TBARS assay (MDA equivalents) in heart homogenates from WT, HET, SMA, HET + ASO, SMA + ASO mice (5 independent experiments; *n* ≥ 4 per group). SMA hearts exhibited markedly elevated TBARS compared with WT and HET, which were restored to WT levels by ASO treatment, while SMA + ASO exhibited significantly decreased TBARS levels compared to the untreated SMA group. All statistical analyses were performed by two‐way ANOVA (factors: genotype and treatment) or one‐way ANOVA (for TBARS), followed by Tukey's *post hoc* test; significance was set at *P* < 0.05. Data are presented as mean ± SD (*n* ≥ 3 biological replicates per group; *P < 0.05, **P < 0.01, ***P < 0.001, ****P < 0.0001; ns, not significant).

Given the pronounced redox defects observed in the heart, we next examined whether SMN‐ASO treatment could restore antioxidant defenses in this tissue (Fig. 7). GSTp, which was reduced in SMA relative to HET, showed a trend toward increase in SMA + ASO, narrowing the difference from HET + ASO, although this change did not reach significance (Fig. 7A,B). GPX4 levels remained significantly lower in SMA than in HET, and SMN‐ASO treatment did not significantly alter GPX4 in SMA + ASO, despite reducing the magnitude of difference relative to HET + ASO (Fig. 7A,B). GRX2 exhibited a similar pattern, without significant SMA versus SMA + ASO differences (Fig. 7A,B).

Despite the limited impact on individual redox enzymes, TBARS analysis revealed complete normalization of lipid peroxidation, with SMA + ASO values reduced to WT levels and SMA versus SMA + ASO difference depicting the significant effect of SMN‐ASO in reducing lipid peroxidation in SMA murine heart (Fig. 7C). These findings suggest that even modest shifts in antioxidant enzyme levels following SMN‐ASO therapy may be sufficient to reduce oxidative damage in the heart, enabling a substantial decrease in lipid peroxidation despite the absence of full GPX4 restoration.

In other organs, ASO treatment elicited modest and tissue‐specific effects (Figs S12–S14). In the brain, no significant changes were detected in GSR, GRX1, or GRX2 (Fig. S12); in the spinal cord, ASO administration did not significantly affect any of the redox enzymes' levels tested (Fig. S13); and in the liver, ASO treatment attenuated the mild SMA‐associated elevations of GRX1 and GRX2 without altering other enzymes (Fig. S14).

Together, these data indicate that SMN‐ASO treatment selectively reduces lipid peroxidation in the heart, while exerting only limited effects on the glutathione‐regulating enzyme network.

## Discussion

**Fig. 8:**
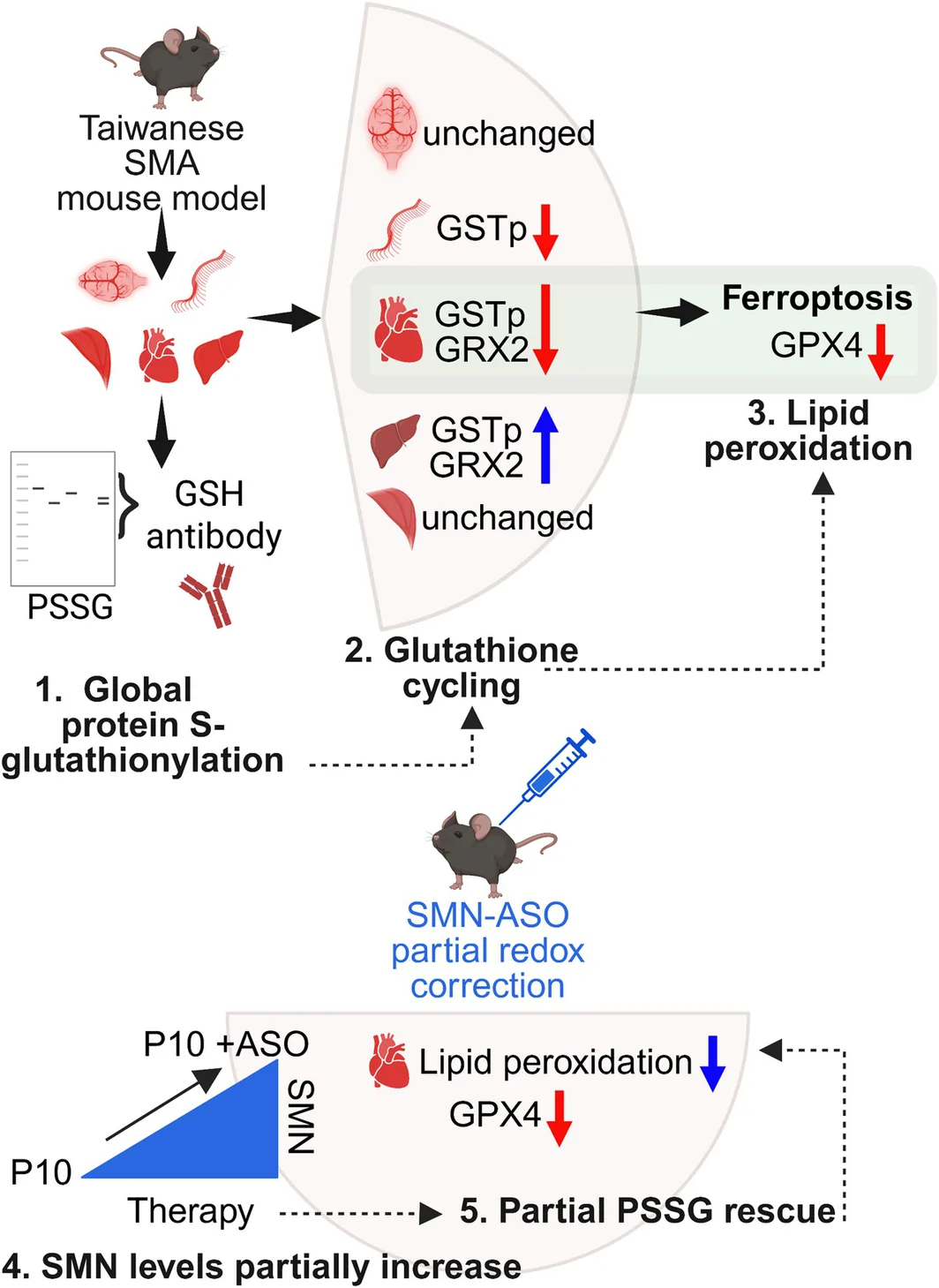
Schematic summary of multi‐organ redox alterations and partial rescue by SMN‐ASO therapy in SMA mice. In the Taiwanese SMA mouse model, organs were harvested at P10 from untreated and SMN‐ASO‐treated mice following tissue homogenization in N‐ethylmaleimide (NEM) buffer to preserve redox state and alkylate free thiols. Non‐reducing western blot analysis with anti‐GSH antibody enabled detection of protein S‐glutathionylation (PSSG) across multiple organs (brain, spinal cord, heart, liver, and gastrocnemius), revealing a global dysregulation of this modification in SMA. These findings prompted characterization of the glutathione‐related redox enzymes (GSR, GSTp, GRX1, and GRX2) to define organ‐specific remodeling. In the brain, all enzymes tested did not show significant differences between the groups. In the spinal cord, SMA showed decreased GSTp levels whereas in the liver both GSTp and GRX2 levels increased. In the gastrocnemius, no major dysregulation was observed either at PSSG or at corresponding redox enzymes. The heart showed the most pronounced imbalance, with elevated GRX1, and reduced GSR, GSTp, GRX2, and GPX4 levels in SMA, accompanied by marked lipid peroxidation detected by TBARS. SMN‐ASO therapy (P1 injection, P10 harvest) restored SMN expression in an organ‐specific manner and partially normalized heart redox enzymes but failed to fully rescue them. Nevertheless, SMN‐ASO treatment markedly mitigated lipid peroxidation in the heart, indicating a partial redox correction driven by improvement of SMN levels. Schematic was created in BioRender. Vrettou, S. (2026) https://BioRender.com/c5hob0j. BioRender publication license: *IW29AWHS2K*.

In this study, we demonstrate that the redox landscape is profoundly disturbed in SMA, characterized by systemic dysregulation of protein S‐glutathionylation and tissue‐specific remodeling of glutathione‐dependent enzymes, culminating in a ferroptotic signature in heart (Fig. 8). By profiling neuronal (brain, spinal cord) and non‐neuronal (heart, liver, gastrocnemius) tissues from Taiwanese SMA mice at early symptomatic (P5) and symptomatic (P10) stages, we show that alterations in the glutathionylated proteome emerge early in a subset of vulnerable tissues and precede enzymatic remodeling in a tissue‐dependent manner. Importantly, coordinated remodeling of glutathione‐regulating enzymes is most pronounced in the heart at the symptomatic stage, whereas other organs exhibit more limited, delayed, or uncoupled changes. These findings highlight that redox dysregulation in SMA does not follow a uniform trajectory across organs but instead reflects intrinsic tissue‐specific redox responses and vulnerabilities.

Notably, heterozygous (*Smn* ^wt^ */* ^ko^; *SMN2* ^*tg/0*^) mice, while often used as phenotypically normal littermate controls, displayed a modest increase in spinal cord PSSG levels at P5. Although clinically unaffected, previous reports indicate that partial SMN reduction can perturb cytoplasmic SMN pools and RNA metabolism and may underlie up to 50% motor neuron attrition [47, 48, 49]. Because spinal motor neurons are highly sensitive to SMN dosage, even moderate depletion may disturb redox equilibrium more readily than in peripheral tissues. These findings support the view that HET mice represent an intermediate molecular phenotype rather than a completely unaffected genotype.

At P5, PSSG depletion occurred without broad changes in enzymes catalyzing this modification, except for GRX2 in heart. This suggests that S‐glutathionylation reflects the integrated tissue redox environment, including GSH/GSSG balance, NADPH flux, and thiol accessibility, rather than enzyme abundance alone [26, 50]. Early loss of PSSG likely represents a rapid redox re‐equilibration driven by transient oxidative shifts, preceding the later adaptive remodeling of the glutathione‐cycling enzymes observed at P10. The heart, with its high mitochondrial and oxidative load, shows the earliest enzymatic adaptation through decreased GRX2, whereas other organs display a temporal lag in which thiol‐redox equilibria shift first and enzyme expression adjusts later [12, 14]. Collectively, these findings reveal that loss of the glutathionylated proteome and remodeling of thiol‐regulating enzymes are temporally coupled yet expressed in a tissue‐specific manner.

By P10, the spinal cord and heart showed the strongest redox remodeling, both with decreased GSTp and additional shifts in cardiac GSR, GRX1, and GRX2. These alterations coincided with GPX4 downregulation and elevated lipid peroxidation in the heart, indicating insufficient detoxification of phospholipid hydroperoxides and a vulnerability to oxidative, rather than inflammatory, lipid damage [51]. Previous studies in SMA reported mitochondrial dysfunction and lipid accumulation [14, 52, 53]. A recent transcriptomic analysis described a broad inflammatory footprint across SMA organs, extending beyond the motor system into multiple organs, yet the upstream metabolic triggers of this inflammation remain incompletely defined [54]. Within this context, the combination of GPX4 loss and elevated lipid peroxidation in SMA hearts is compatible with a ferroptosis‐like stress [51] in SMA hearts, potentially linking redox‐metabolic imbalance to tissue‐specific inflammatory signaling. While GPX4 has not previously been examined in SMA, its marked loss in the heart, but not in the spinal cord, indicates that distinct cell death pathways may operate across organs. Although the mode of cell death triggered by SMN depletion remains a matter of debate, with apoptosis, autophagy, and necroptosis having been proposed [55, 56, 57, 58, 59], our findings indicate that ferroptosis may contribute specifically to cardiac pathology. This aligns with congenital heart abnormalities reported in SMA patients and mouse models [31, 60, 61].

The dysregulation of enzymes governing S‐glutathionylation cycling provides mechanistic insight into this redox vulnerability. GRX1 and GRX2 catalyze deglutathionylation, whereas GSR and GSTp sustain the reducing environment required for dynamic thiol modification [45]. Previous reviews and proteomic studies have shown that SMN depletion alters cellular redox regulation. Zilio *et al*. [17] summarized oxidative stress and mitochondrial dysfunction as central features of SMA and reported alterations in multiple antioxidant enzymes, including superoxide dismutases, catalase, and glutathione peroxidases. Consistent with this, Thelen *et al*. [25] identified GRX2 as upregulated in SMA motor neurons, suggesting compensatory attempts to counteract oxidative stress. The organ‐specific defects we observed at P10 further highlight differential redox plasticity: the liver showed increased GSTp and mitochondrial GRX2 despite unchanged GSR, consistent with adaptive reinforcement of thiol cycling, whereas the heart exhibited parallel reductions in GSTp and GRX2 alongside elevated lipid peroxidation, indicating diminished redox buffering and impaired mitochondrial control.

The divergent remodeling of glutathione‐related enzymes across organs likely reflects a combination of intrinsic metabolic demand, SMN‐dependent transcript regulation, and tissue‐specific redox buffering capacity. As reviewed by Zilio *et al*. [17] several transcripts encoding antioxidant and mitochondrial enzymes, including GSR, GPX, GRX, and GST family members, are consistently altered in SMA models. High‐energy organs such as heart, with dense mitochondria and sustained oxidative flux, have a greater demand for continuous GSH regeneration, predisposing them to early redox disequilibrium [14, 52]. In contrast, the brain appears comparatively resilient, consistent with lower baseline ROS generation and efficient astrocyte‐neuron coupling that stabilizes the local GSH pool [62]. The spinal cord displays an intermediate profile, in which selective GSTp loss may signal local depletion of cytosolic GSH rather than global redox collapse. Together, these features delineate a hierarchy of oxidative susceptibility shaped by both SMN‐dependent transcriptional control and tissue metabolic context.

SMN‐ASO therapy, although effective in elevating SMN expression, exerted selective and tissue‐dependent effects on redox homeostasis. Across organs, SMN‐ASO modestly increased SMN but did not restore the glutathione‐regulating enzyme network or normalize GPX4 levels. In the heart, SMN‐ASO induced a modest upward shift in GSTp but did not significantly alter GRX2 or GPX4 levels, indicating that enzymatic remodeling remained largely uncorrected. Notably, despite this limited enzyme response, lipid peroxidation was markedly reduced and approached WT values following SMN‐ASO treatment. These findings indicate that mitigation of oxidative damage can occur independently of full restoration of glutathione‐cycling enzymes, highlighting an uncoupling between SMN‐dependent modulation of redox stress and normalization of the underlying enzymatic machinery. This interpretation is consistent with prior studies showing that SMN restoration can alleviate oxidative stress and mitochondrial dysfunction in SMA tissues and neurons through multiple, context‐dependent pathways [12, 25].

Our findings extend earlier reports describing oxidative stress and mitochondrial dysfunction as recurring features in SMA pathogenesis [11, 25, 63, 64]. Previous antioxidant treatments, such as *N*‐acetylcysteine or pyruvate supplementation, enhanced SMN translation in neurons but did not evaluate thiol‐based modifications systemically [25]. By directly analyzing S‐glutathionylation, we demonstrate that thiol redox signaling is broadly perturbed across SMA organs, establishing S‐glutathionylation as a sensitive molecular footprint of redox imbalance rather than a readout of irreversible oxidative damage. However, extension of this framework to the glutathione‐glutaredoxin axis and GPX4 reveals a marked organ specificity, with coordinated enzymatic remodeling and ferroptosis‐associated vulnerability emerging predominantly in the heart. These findings indicate that while redox disequilibrium is systemic in SMA, its downstream enzymatic and ferroptotic consequences are shaped by tissue‐intrinsic redox architecture and may further reflect differences in oxidative versus nitrosative stress burden [65, 66], mitochondrial reliance [67], protein turnover [68], or redox‐buffering capacity across organs [69].

SMA is increasingly recognized as a multisystem disorder affecting motor neurons and peripheral organs including heart, liver, and skeletal muscle [20]. The widespread S‐glutathionylation dysregulation observed here supports this systemic view and is consistent with disruption of redox sensing rather than uniform oxidative damage [69]. Reduced PSSG levels likely denote impaired thiol‐based redox sensing and loss of adaptive redox control, which can precede or occur independently of downstream redox signaling responses such as enzymatic remodeling. As proposed by Jones, distinct tissues operate at different redox set points and engage redox cell signaling only when specific thresholds are exceeded, providing a framework to explain why PSSG alterations do not uniformly translate into coordinated enzymatic responses across organs [69].

Recent studies further highlight the promise and limitations of antioxidant therapy in SMA. Prenatal or early postnatal treatment with the natural antioxidant ergothioneine (ERGO) improved survival, motor performance, and oxidative stress markers in SMNΔ7 mice [70]. However, ERGO failed to restore mitochondrial biogenesis, consistent with the fact that ERGO does not activate PGC1aThis finding suggests that antioxidant therapy alone may not fully correct mitochondrial dysfunction or the broader metabolic consequences of SMN deficiency.

A complementary line of evidence comes from a recent study using erastin‐treated cell lines and acetaminophen‐induced liver injury models, which showed that protein S‐glutathionylation protects against ferroptosis induced by glutathione depletion, mechanistically linking thiol redox cycling to ferroptotic resistance [71]. In the context of our study, SMA hearts exhibit marked GPX4 loss and elevated lipid peroxidation, consistent with heightened ferroptotic vulnerability [51], whereas SMN‐ASO treatment reduces lipid peroxidation; restoration of glutathione‐regulated enzymes is incomplete. While the aforementioned studies directly assessed ferroptotic sensitivity using pharmacological induction and cell death readouts [71], our work complements this framework by identifying endogenous, tissue‐specific redox and GPX4 alterations *in vivo* that define a permissive landscape for ferroptotic stress in heart rather than demonstrating ferroptosis *per se*.

Our study has several limitations. Whole‐tissue lysates were analyzed, which capture average redox profiles but obscure potential cell‐type‐specific differences. Antibody‐based detection of total S‐glutathionylation, though validated under reducing and alkylating conditions, provides semi‐quantitative resolution compared with proteomics approaches. Moreover, the modest ASO‐induced redox effects in spinal cord indicate that additional mechanisms may modulate tissue responsiveness. This study was not designed to assess survival or motor outcomes. However, previous reports using comparable systemic neonatal ASO regimens in the Taiwanese SMA line have demonstrated partial phenotypic improvement and lifespan extension [38, 41].

Collectively, our findings reveal that tissue‐specific remodeling of the glutathione and S‐glutathionylation system provides a mechanistic basis for the differential vulnerability of organs in SMA. The persistence of redox imbalances despite partial SMN restoration underscores that metabolic resilience and antioxidant capacity are not uniformly governed by SMN levels alone but are shaped by organ‐intrinsic redox architecture and context‐dependent engagement of downstream redox signaling pathways. While protein S‐glutathionylation serves as a sensitive footprint of redox sensing, our data demonstrate that it is insufficient on its own to infer tissue impairment at the level of enzymatic regulation or ferroptotic susceptibility, which instead require deeper, layered characterization of the redox and metabolic network. By integrating S‐glutathionylation with analysis of glutathione‐regulating enzymes and lipid peroxidation, our study identifies the heart as a particularly redox‐vulnerable organ in SMA. Together, these data broaden the mechanistic landscape of SMA beyond RNA metabolism and motor neuron loss, positioning glutathione metabolism and S‐glutathionylation dynamics as central but context‐dependent components of disease pathology. Accordingly, therapeutic strategies combining SMN‐directed correction with tissue‐informed redox‐ or ferroptosis‐modulating interventions may be required to more effectively preserve systemic organ function in SMA.

## Author contributions

SV was involved in conceptualization, methodology, investigation, data curation, formal analysis, writing original draft. BW was involved in conceptualization, writing – review and editing, funding acquisition and supervision.

## Supporting information


**Fig. S1.** Protein S‐glutathionylation (PSSG) in liver and gastrocnemius at early symptomatic stage postnatal day 5 (P5).
**Fig. S2.** Global slopegraph summary of protein S‐glutathionylation (PSSG) at P5 across all organs.
**Fig. S3.** Protein S‐glutathionylation (PSSG) in liver and gastrocnemius at late symptomatic stage P10.
**Fig. S4.** Global slopegraph summary of protein S‐glutathionylation (PSSG) at P10 across all organs.
**Fig. S5.** Expression of glutathione‐related redox enzymes in brain at P5.
**Fig. S6.** Expression of glutathione‐related redox enzymes in spinal cord and heart at P5.
**Fig. S7.** Lipid peroxidation levels in brain, spinal cord, and heart at P10.
**Fig. S8.** Expression of glutathione‐related redox enzymes in liver and gastrocnemius at P10.
**Fig. S9.** SMN protein levels in brain, spinal cord, and heart of ASO‐treated and untreated mice at P10.
**Fig. S10.** SMN protein levels in liver and gastrocnemius of ASO‐treated and untreated mice at P10.
**Fig. S11.** Protein S‐glutathionylation (PSSG) in liver and gastrocnemius of ASO‐treated and untreated mice at P10.
**Fig. S12.** Expression of glutathione‐related redox enzymes in brain of ASO‐treated and untreated mice at P10.
**Fig. S13.** Expression of glutathione‐related redox enzymes in spinal cord of ASO‐treated and untreated mice at P10.
**Fig. S14.** Expression of glutathione‐related redox enzymes in liver of ASO‐treated and untreated mice at P10.

## Data Availability

The datasets generated and analyzed during this study are available in the Dryad Digital Repository at: https://doi.org/10.5061/dryad.3xsj3txw1.
