# Supplementary material for: Organ‐specific redox imbalances in spinal muscular atrophy mice are partially rescued by SMN antisense oligonucleotides
Source: FEBS Lett. 2026 Feb 13;600(16):2262–82. doi: 10.1002/1873-3468.70303 (PMC13502620; doi:10.1002/1873-3468.70303)
Supplement: Supplementary file 1 [file FEB2-600-2262-s001.pdf]

Supplementary Information

Title:
Organ-specific redox imbalances in spinal muscular atrophy mice are partially rescued by SMN antisense oligonucleotides

Authors:
Sofia Vrettou [1,2], Brunhilde Wirth[1,2,3]
[1]Institute of Human Genetics, University Hospital of Cologne, University of Cologne, 50931 Cologne, Germany.
[2]Center for Molecular Medicine Cologne, University of Cologne, 50931 Cologne, Germany.
[3]Center for Rare Diseases, University Hospital of Cologne, University of Cologne, 50931 Cologne, Germany.
Corresponding authors: sofia.vrettou@uk-koeln.de, brunhilde.wirth@uk-koeln.de

Contents:
S1. Protein S-glutathionylation (PSSG) in liver and gastrocnemius at early symptomatic stage postnatal day 5 (P5).
S2. Global slopegraph summary of protein S-glutathionylation (PSSG) at P5 across all organs.
S3. Protein S-glutathionylation (PSSG) in liver and gastrocnemius at late symptomatic stage P10.
S4. Global slopegraph summary of protein S-glutathionylation (PSSG) at P10 across all organs.
S5. Expression of glutathione-related redox enzymes in brain at P5.
S6. Expression of glutathione-related redox enzymes in spinal cord and heart at P5.
S7. Lipid peroxidation levels in brain, spinal cord, and heart at P10.
S8. Expression of glutathione-related redox enzymes in liver and gastrocnemius at P10.
S9. SMN protein levels in brain, spinal cord, and heart of ASO-treated and untreated mice at P10.
S10. SMN protein levels in liver and gastrocnemius of ASO-treated and untreated mice at P10.
S11. Protein S-glutathionylation (PSSG) in liver and gastrocnemius of ASO-treated and untreated mice at P10.
S12. Expression of glutathione-related redox enzymes in brain of ASO-treated and untreated mice at P10.
S13. Expression of glutathione-related redox enzymes in spinal cord of ASO-treated and untreated mice at P10.
S14. Expression of glutathione-related redox enzymes in liver of ASO-treated and untreated mice at P10.

Note:
Supplementary Figures S1–S14 correspond to Figures 1–8 in the main manuscript. All blots were derived from independent biological replicates and exported at ≥ 300 dpi for optimal clarity.

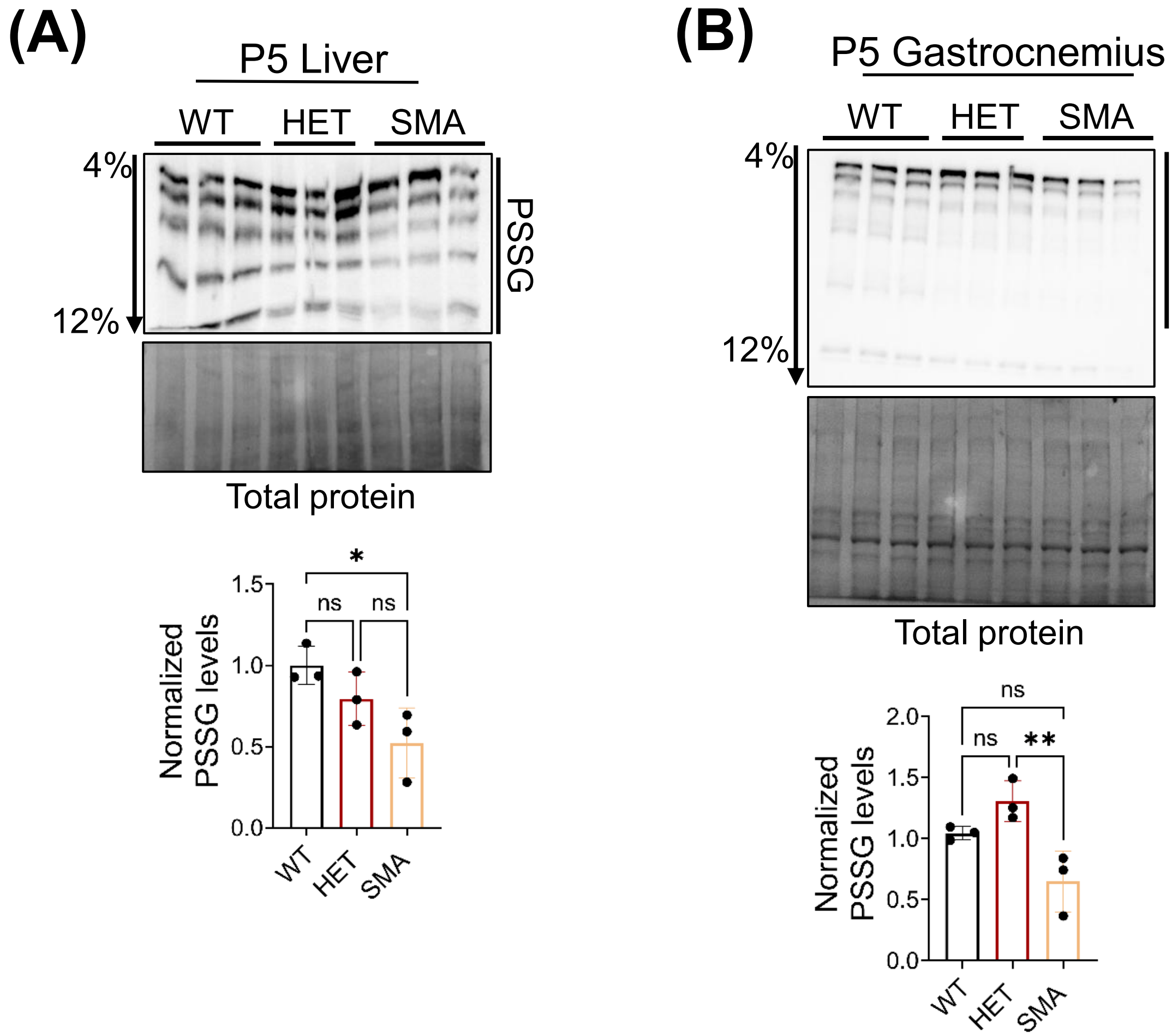

Supplementary Figure S1. Protein S-glutathionylation (PSSG) in liver and gastrocnemius at early symptomatic stage postnatal day 5 (P5).

(A) Representative non-reducing Western blot showing PSSG in liver protein lysates from WT, HET, and SMA mice at P5, with corresponding quantification normalized to total protein. SMA liver showed a reduction compared to WT, whereas WT and HET did not differ significantly.

(B) Representative non-reducing Western blot showing PSSG in gastrocnemius protein lysates from WT, HET, and SMA mice at P5, with corresponding quantification normalized to total protein. SMA gastrocnemius displayed a significant reduction versus HET but not WT, while WT and HET were not significantly different.

Total protein reverse staining served as loading control. Data were analyzed by one-way ANOVA (factor:genotype) with Tukey's post-hoc test. Bars show mean ± SD with n = 3 biological replicates per group. Significance thresholds: *$p < 0.05$, **$p < 0.01$, ns denotes not significant.

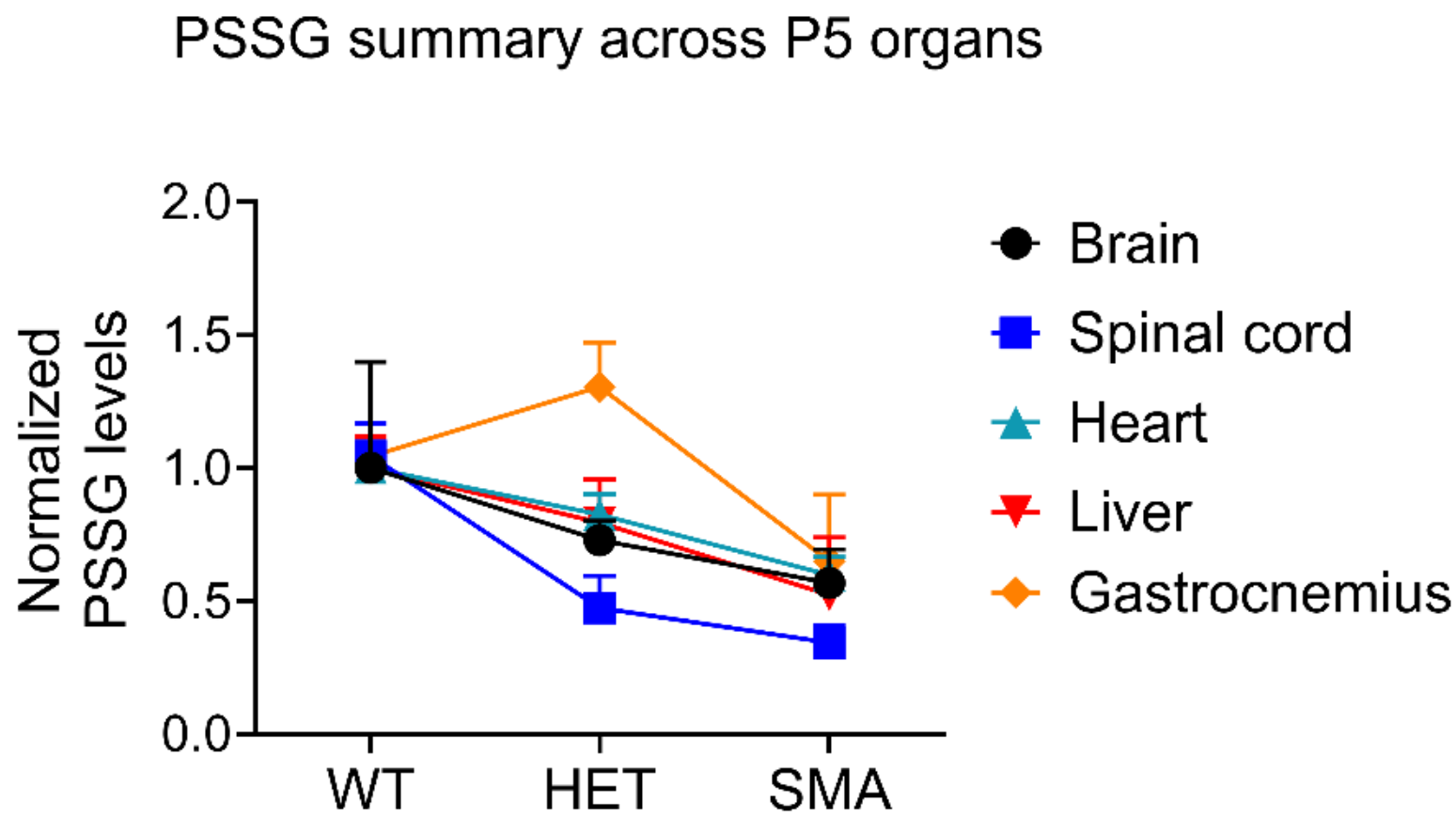

Supplementary Figure S2. Global slopegraph summary of protein S-glutathionylation (PSSG) at postnatal day 5 (P5) across all organs.
Summary slopegraph of PSSG levels across all five organs (brain, spinal cord, heart, liver, and gastrocnemius) at P5. Overall, all organs showed a downward trend from WT to SMA. Spinal cord and heart exhibited the strongest early decreases, liver displayed a moderate reduction relative to WT, brain showed a mild decline, and gastrocnemius presented a drop from HET to SMA.
Symbols represent mean values for each organ and genotype, and error bars indicate the standard deviation derived from individual Western blot measurements.

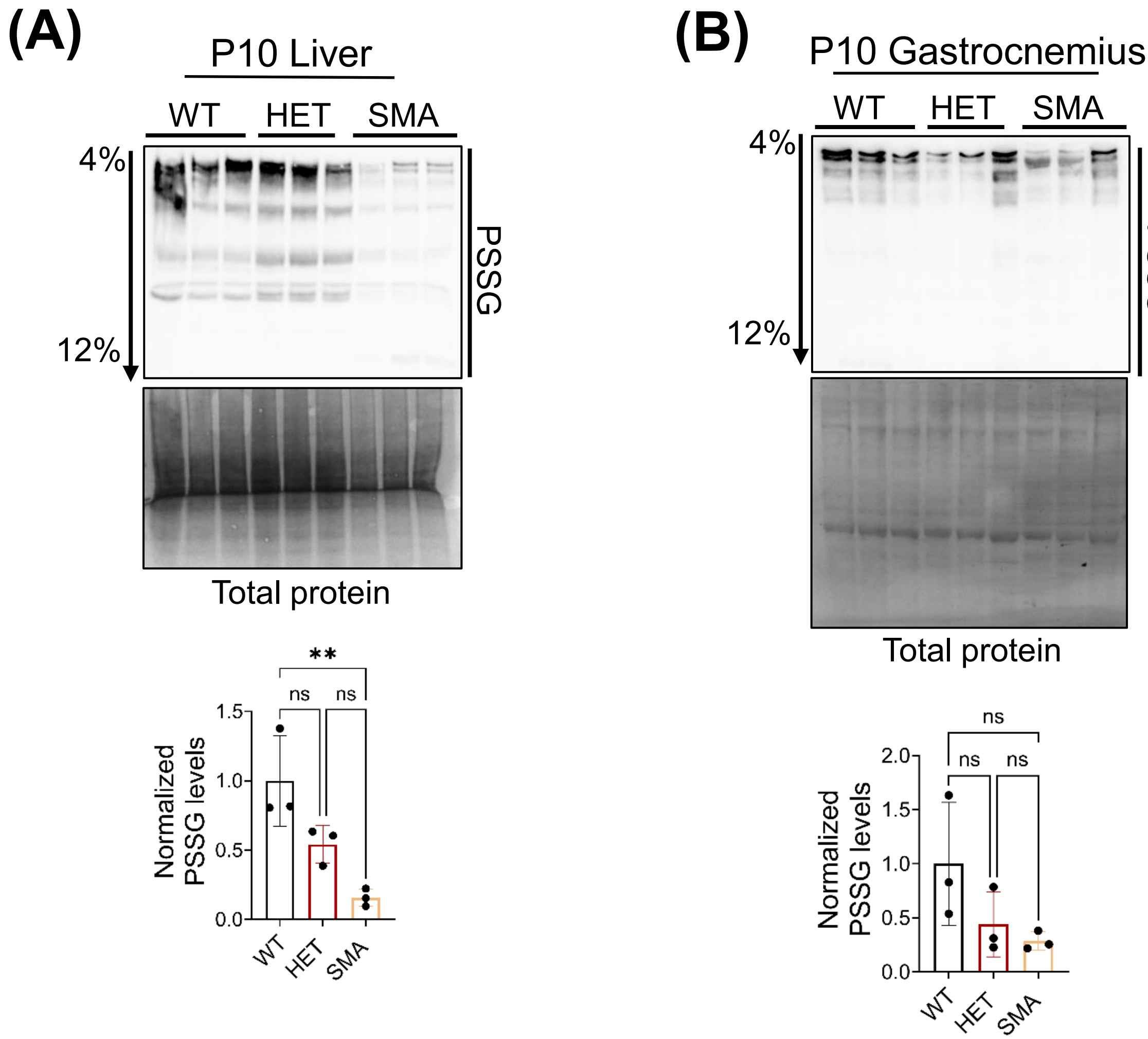

Supplementary Figure S3. Protein S-glutathionylation (PSSG) in liver and gastrocnemius at late symptomatic stage 10 (P10).
(A) Representative non-reducing Western blot of PSSG in liver lysates from WT, HET, and SMA mice at P10, with quantification normalized to total protein. SMA liver displayed significantly reduced PSSG levels compared to WT but not HET. No significant differences were observed amongst WT and HET.
(B) Representative non-reducing Western blot of PSSG in gastrocnemius lysates from WT, HET, and SMA mice at P10, with quantification normalized to total protein. No significant differences were observed among genotypes.
Total protein reverse staining served as loading control. One-way ANOVA (factor:genotype) followed by Tukey‘s post-hoc test for all pairwise comparisons. Bars show mean ±SD with n≥3 biological replicates per group. Significance thresholds: $^{**}p < 0.01$, ns denotes not significant.

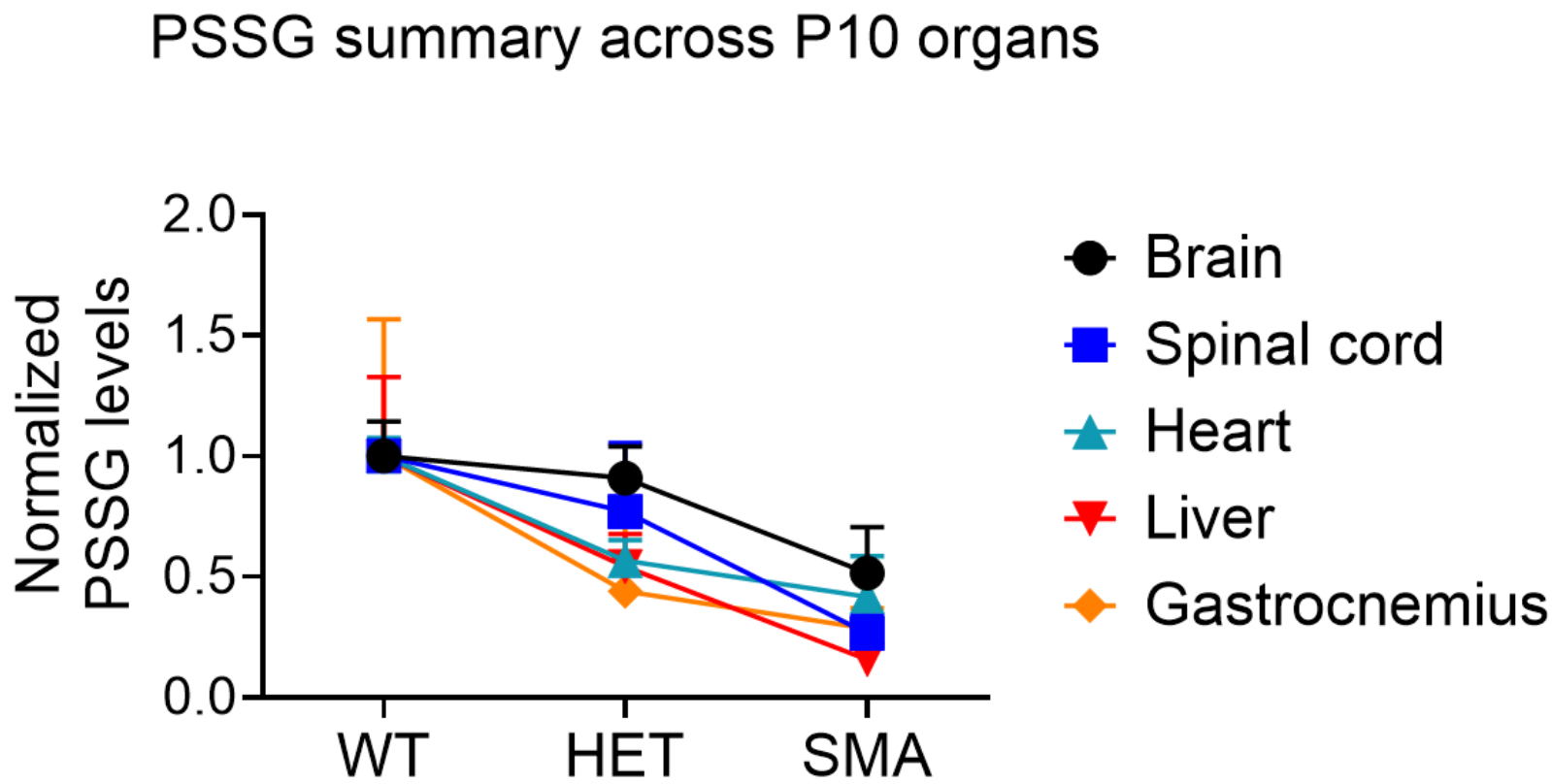

Supplementary Figure S4. Global slopegraph summary of protein S-glutathionylation (PSSG) at postnatal day 10 (P10) across all organs.

Summary slopegraph illustrating normalized PSSG levels across all five organs (brain, spinal cord, heart, liver, and gastrocnemius) at P10. Overall all organs showed a downward trend from WT to SMA. Spinal cord and heart exhibited pronounced decreases, liver showed the most consistent reduction, brain displayed a mild decline, and gastrocnemius changes were not statistically significant.

Symbols represent mean values for each organ and genotype, and error bars indicate the standard deviation derived from individual Western blot measurements.

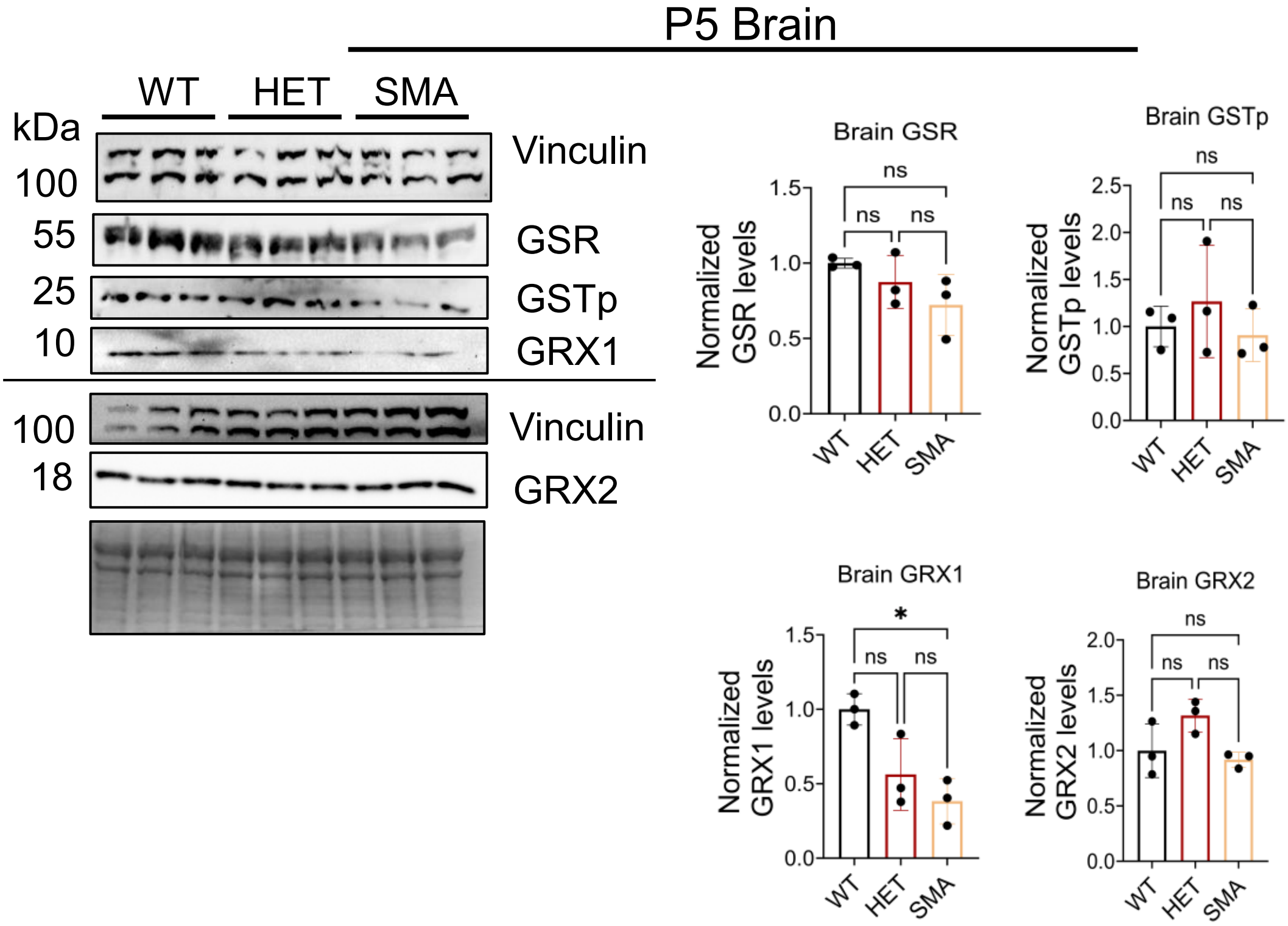

Supplementary Figure S5. Expression of glutathione-related redox enzymes in brain at postnatal day 5 (P5).
Representative Western blots and quantification of redox enzymes in brain lysates from WT, HET, and SMA mice at P5. Glutathione reductase (GSR), glutathione-S-transferase p (GSTp), glutaredoxin-2 (GRX2), and glutaredoxin-1 (GRX1 were analyzed by Western blot, normalized to vinculin, and expressed relative to WT. SMA brains showed a significant reduction in GRX1 levels compared to WT, whereas GSR, GSTp, and GRX2 remained unchanged among genotypes.
One-way ANOVA (factor:genotype) followed by Tukey‘s post-hoc test for all pairwise comparisons. Bars represent mean ±SD of 3 biological replicates per group. Significance thresholds: *p < 0.05, ns denotes not significant.

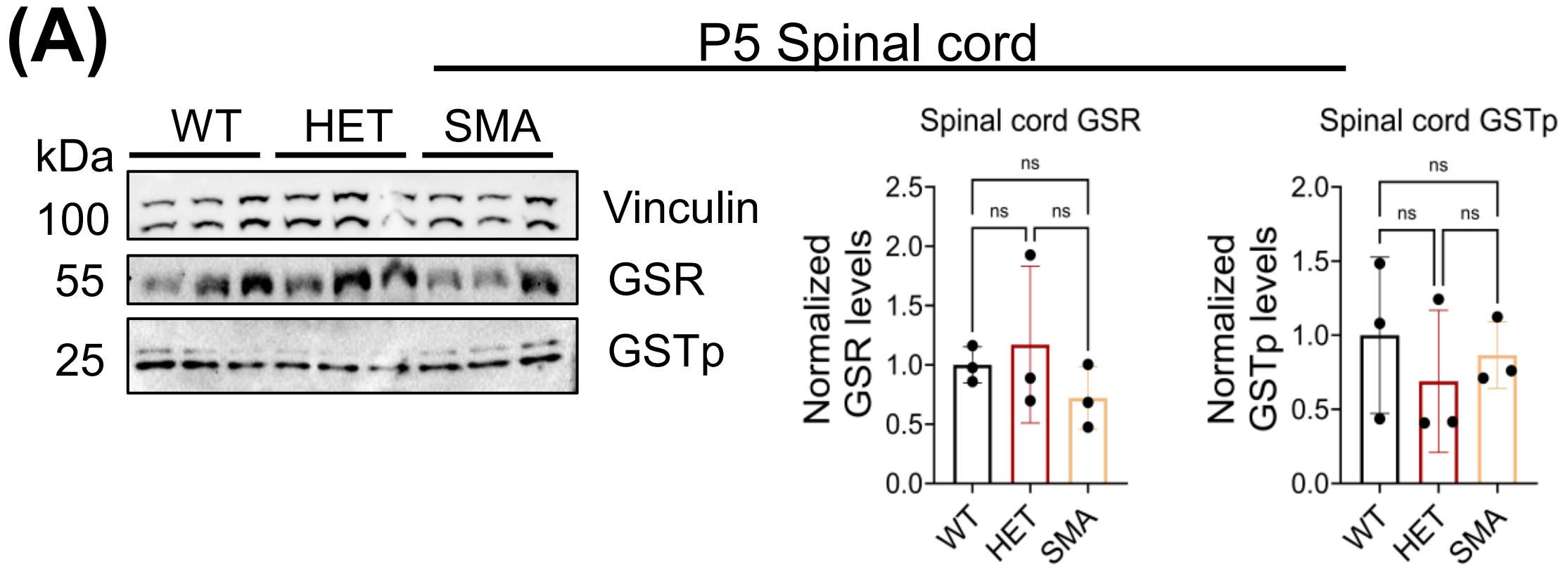

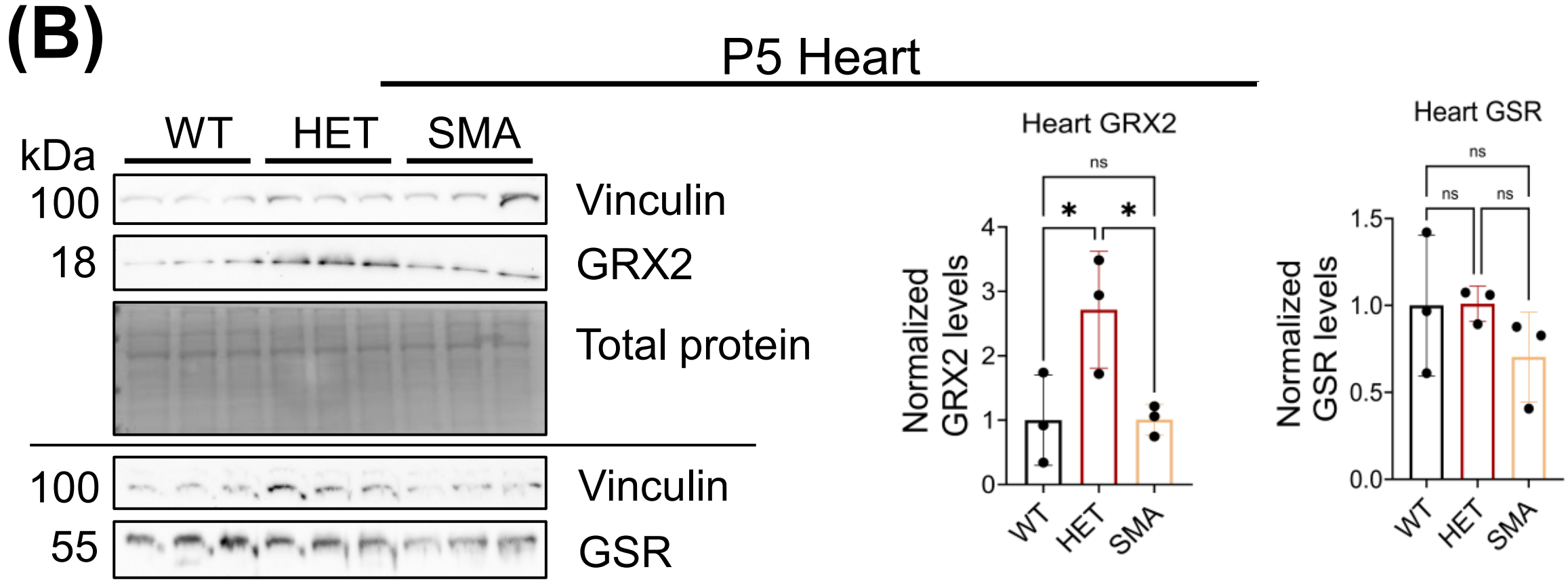

Supplementary Figure S6. Expression of glutathione-related redox enzymes in spinal cord and heart at postnatal day 5 (P5).
(A) Representative Western blots and quantification of glutathione reductase (GSR) and glutathione-S-transferase p (GSTp) in spinal cord lysates from WT, HET, and SMA mice at P5. No significant differences were detected among genotypes for either enzyme.
(B) Representative Western blots and quantification of glutathione reductase (GSR) and glutaredoxin-2 (GRX2) in heart lysates from WT, HET, and SMA mice at P5. GRX2 levels were significantly increased in HET hearts compared to WT and decreased in SMA compared to HET, whereas GSR levels remained unchanged. Total protein staining served as the loading control for the GRX2 blot.
One-way ANOVA (factor:genotype) followed by Tukey's post-hoc test for all pairwise comparisons. Bars represent mean ±SD of 3 biological replicates per group. Significance thresholds: $^*p < 0.05$, ns denotes not significant.

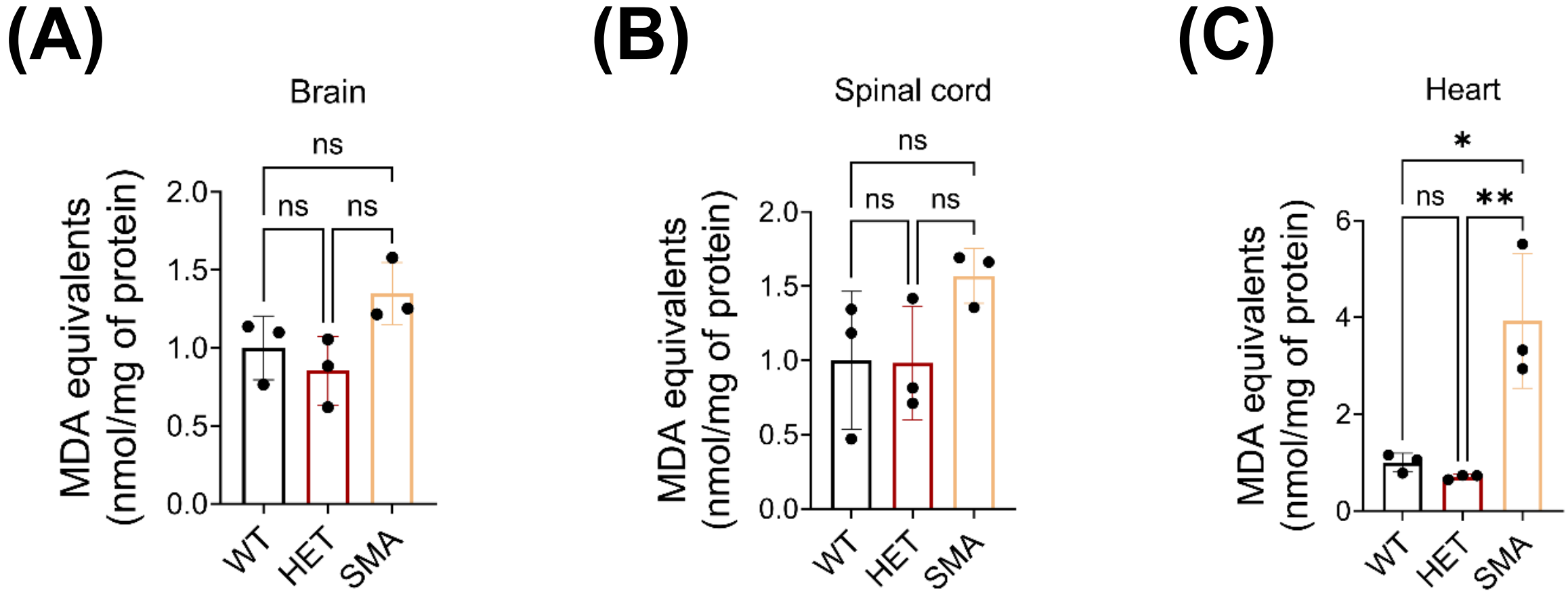

Supplementary Figure S7. Lipid peroxidation levels in brain, spinal cord, and heart at postnatal day 10 (P10).

(A-C)Thiobarbituric acid reactive substances (TBARS) levels were measured as an index of lipid peroxidation in brain (A), spinal cord (B), and heart (C) whole tissue homogenates from WT, HET, and SMA mice at P10. Values are expressed as nanomoles (nmol) of malondialdehyde (MDA) per milligram (mg) of protein and normalized to the WT group mean. Amongst the analyzed organs, only SMA hearts showed a significant increase in TBARS levels compared to both WT and HET, whereas no significant differences were detected in brain or spinal cord.

One-way ANOVA (factor:genotype) followed by Tukey's post-hoc test for all pairwise comparisons. Bars represent mean ±SD of 3 biological replicates per group. Significance thresholds: *$p < 0.05$, **$p < 0.01$, ns denotes not significant.

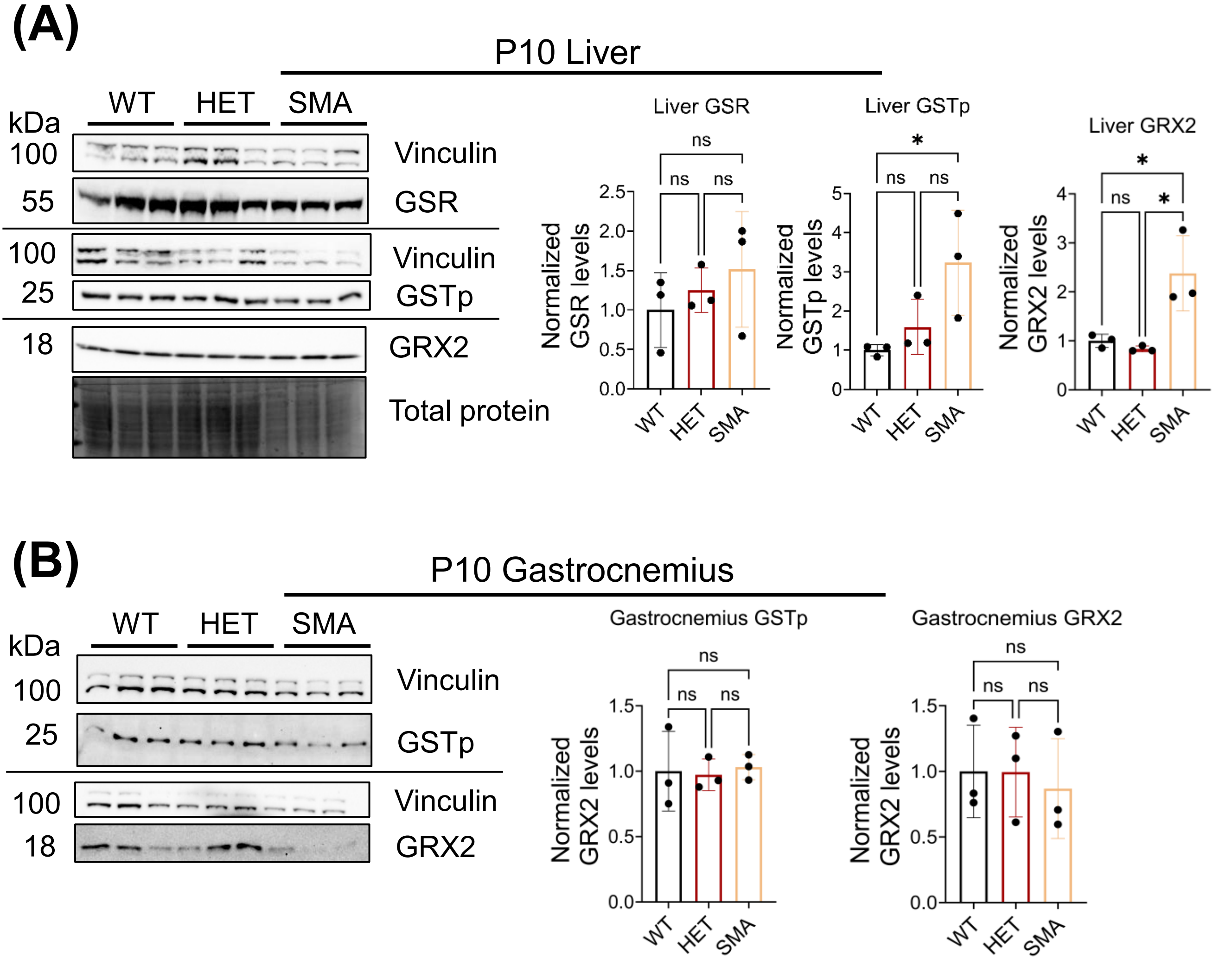

Supplementary Figure S8: Expression of glutathione-related redox enzymes in liver and gastrocnemius at postnatal day 10 (P10).
(A) Representative Western blots and quantification of glutathione reductase (GSR), glutathione-S-transferase p (GSTp), and glutaredoxin 2 (GRX2) in liver lysates from WT, HET, and SMA mice at P10. SMA livers showed significantly increased GSTp and GRX2 levels compared to WT, whereas GSR levels remained unchanged.
(B) Representative Western blots and quantification of GSTp and glutaredoxins-2 (GRX2) in gastrocnemius lysates from WT, HET, and SMA mice at P10. No significant differences were observed among genotypes for either enzyme.
One-way ANOVA (factor:genotype) followed by Tukey's post-hoc test for all pairwise comparisons. Bars represent mean ±SD of 3 biological replicates per group. Significance thresholds: *p < 0.05, ns denotes not significant.

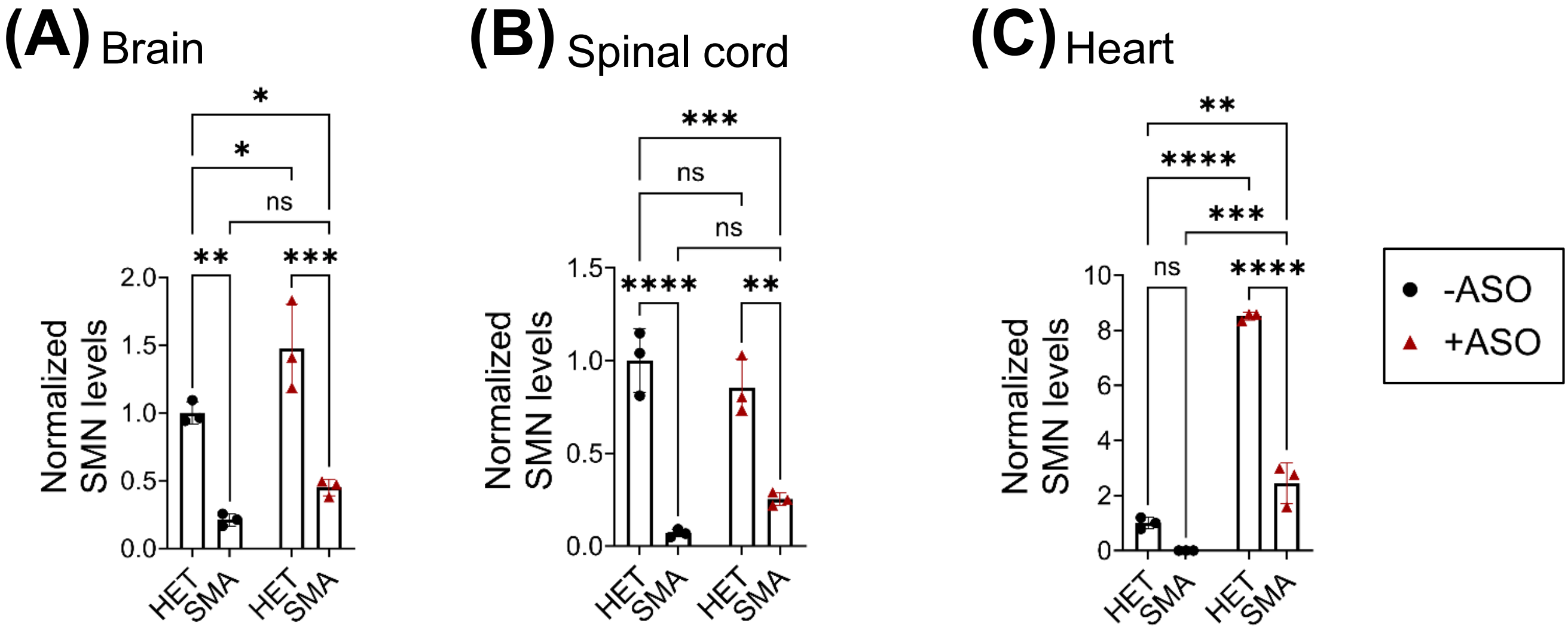

Supplementary Figure S9. SMN protein levels quantification in brain, spinal cord, and heart of ASO-treated and untreated SMA and HET mice at postnatal day 10 (P10).

(A-C) Quantification of SMN protein levels in brain (A), spinal cord (B), and heart (C) from HET and SMA mice injected with antisense oligonucleotides (ASO; +ASO) or untreated (-ASO). SMN levels were measured by Western blot, normalized to vinculin or toatal protein reverse staining, and expressed relative to the mean of untreated HET. These data correspond to the same blots used for the summary graph in the main Figure 5, here expanded to show full pairwise comparisons including HET groups.

(A) In the brain, ASO treatment led to a further increase in HET+ASO compared to HET, resulting in a larger difference between HET+ASO and SMA+ASO, whereas no significant difference was detected between SMA and SMA+ASO.

(B) In the spinal cord, the difference between HET and SMA was reduced in ASO-treated groups, likely due to a mild increase in SMN in SMN+ASO, as reflected by the smaller gap between HET and SMA+ASO compared to HET and SMA; however, SMA vs SMA+ASO remained not significant.

(C) In the heart, ASO treatment significantly increased SMN levels in SMA+ASO compared to SMA, and HET+ASO hearts also showed a strong elevation compared to untreated HET.

Two-way ANOVA (factors:genotype and treatment) followed by Tukey‘s post-hoc test for all pairwise comparisons. Bars represent mean ±SD of 3 biological replicates per group. Significance thresholds: *$p < 0.05$, **$p < 0.01$, ***$p < 0.001$, ****$p < 0.0001$; ns, not significant.

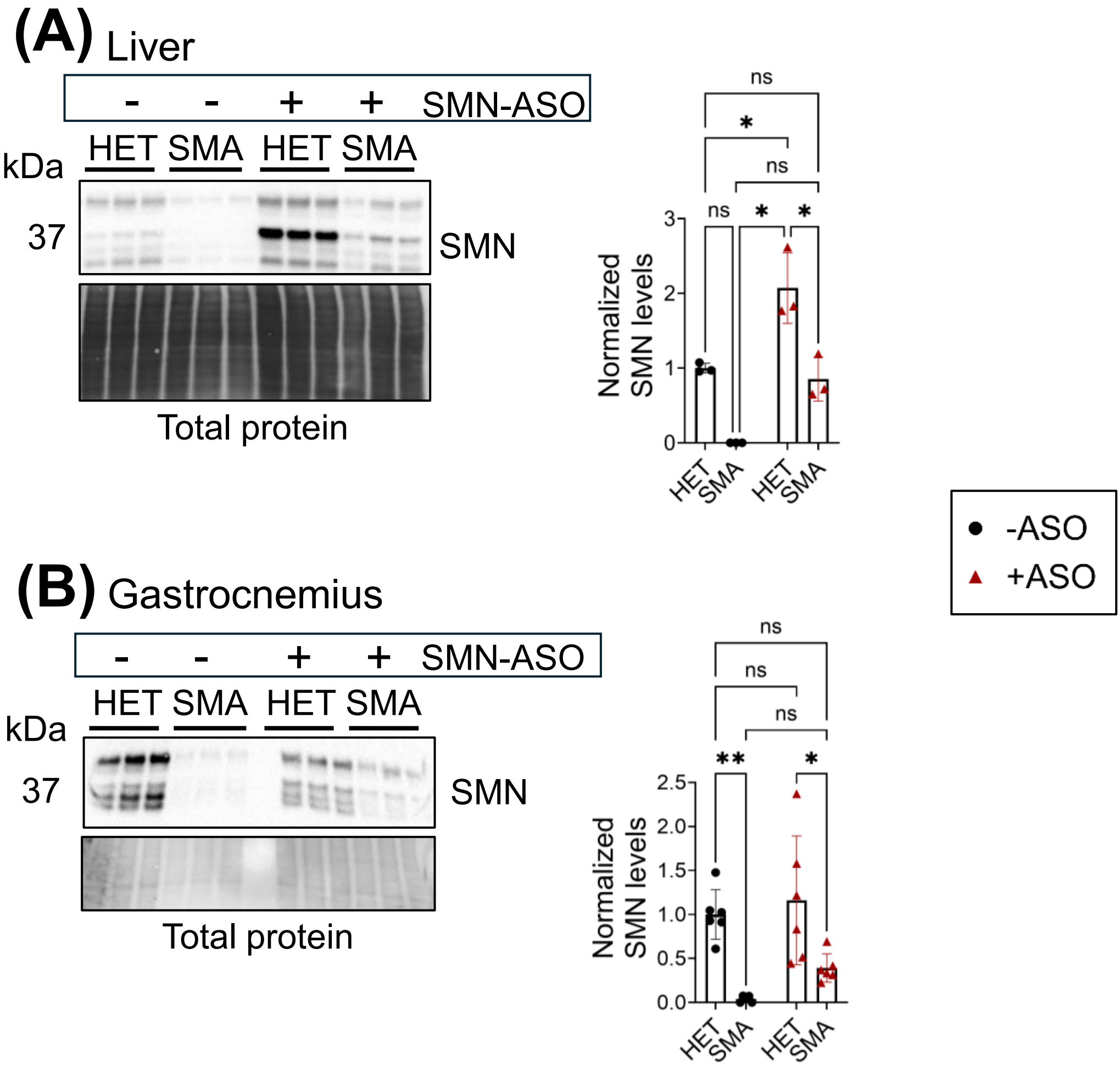

Supplementary Figure S10. SMN protein levels in liver and gastrocnemius of ASO-treated and untreated SMA and HET mice at postnatal day 10 (P10).

(A) Representative Western blots and quantification of SMN protein levels in liver lysates from HET and SMA mice injected with antisense oligonucleotides (ASO; +ASO) or untreated (-ASO). SMN-ASO led to a modest increase in SMA+ASO but not rendered it significant versus the untreated SMA group, whereas HET+ASO SMN levels were significantly increased.

(B) Representative Western blots and quantification of SMN protein levels in gastrocnemius lysates from the same experimental groups. HET+ASO showed significantly higher SMN levels compared to SMA+ASO, but the gap was mitigated as it compares to HET vs SMA SMN levels. Two-way ANOVA (factors:genotype and treatment) followed by Tukey's post-hoc test for all pairwise comparisons. Bars represent mean ±SD of at least 3 biological replicates for all groups. Significance thresholds: $*p < 0.05$, $**p < 0.01$, ns denotes not significant.

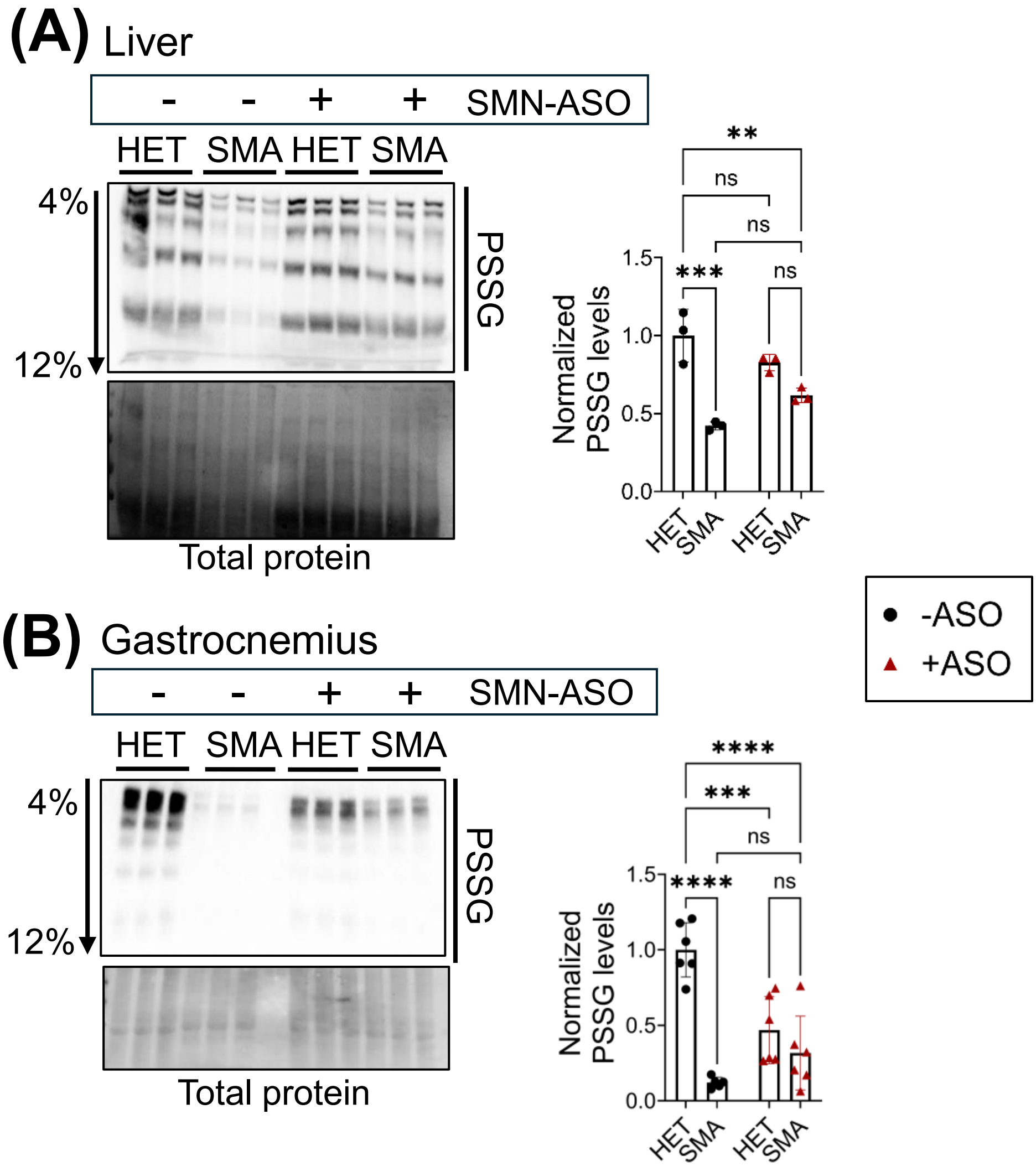

Supplementary Figure S11. Protein S-glutathionylation (PSSG) in liver and gastrocnemius of ASO-treated and untreated SMA and HET mice at postnatal day 10 (P10).

(A-B) Representative non-reducing Western blots and quantification of PSSG normalized to total protein in liver (A) and gastrocnemius (B). In both organs, SMA showed reduced PSSG compared to HET, while SMA+ASO exhibited partial restoration toward HET+ASO levels without significant difference from SMA. Total protein reverse staining served as loading control.

Two-way ANOVA (factors:genotype and treatment) followed by Tukey's post-hoc test for all pairwise comparisons. Bars represent mean ±SD of at least 3 biological replicates for all groups. Significance thresholds: **p < 0.01, ***p < 0.001, ****p < 0.0001; ns, not significant.

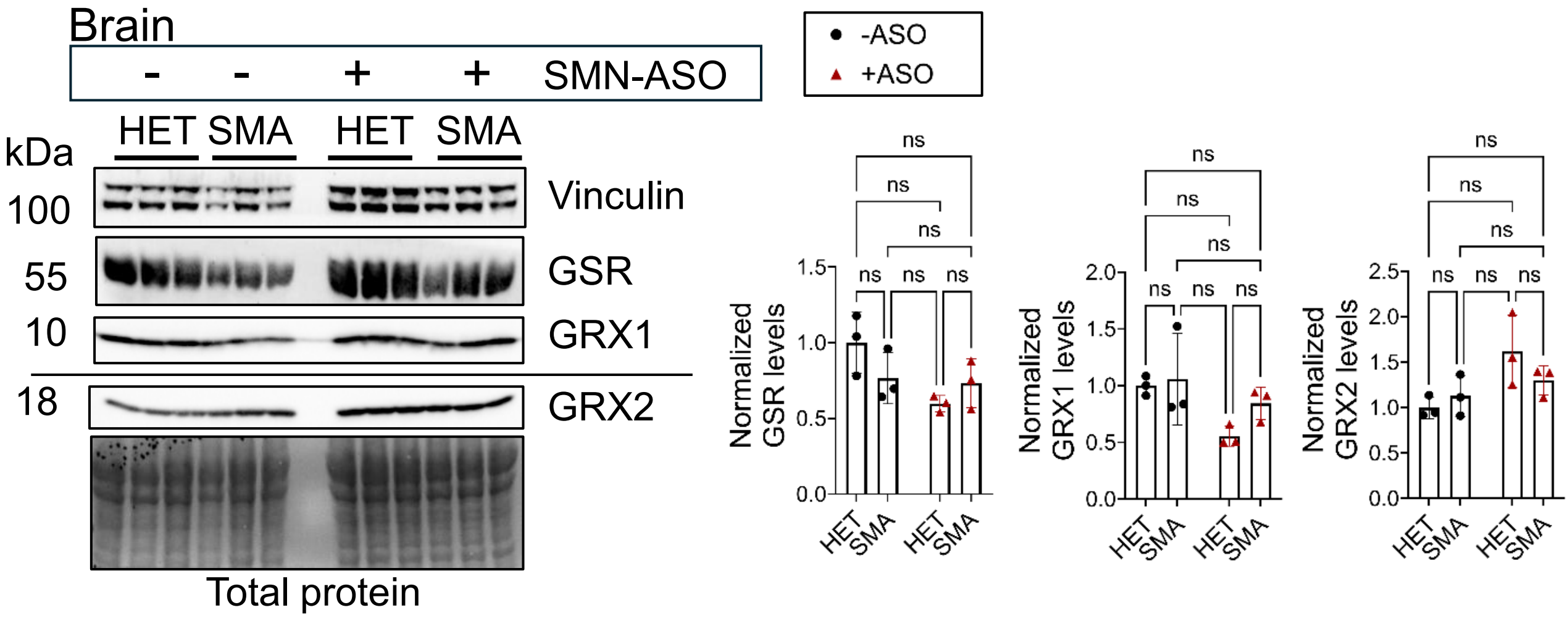

Supplementary Figure S12. Expression of glutathione-related redox enzymes in brain of ASO-treated and untreated SMA and HET mice at postnatal day 10 (P10). Representative Western blots and quantification of glutathione reductase (GSR), glutaredoxin-1 and 2 (GRX1, GRX2) in brain lysates from HET and SMA mice injected with antisense oligonucleotides (ASO; +ASO) or untreated (-ASO). All enzymes were normalized to vinculin or total protein, and expressed relative to untreated HET. No significant differences were detected between genotypes or treatment groups for any of the analyzed enzymes.

Two-way ANOVA (factors:genotype and treatment) followed by Tukey‘s post-hoc test for all pairwise comparisons. Bars represent mean ±SD of 3 biological replicates for all groups. Significance thresholds: ns, not significant.

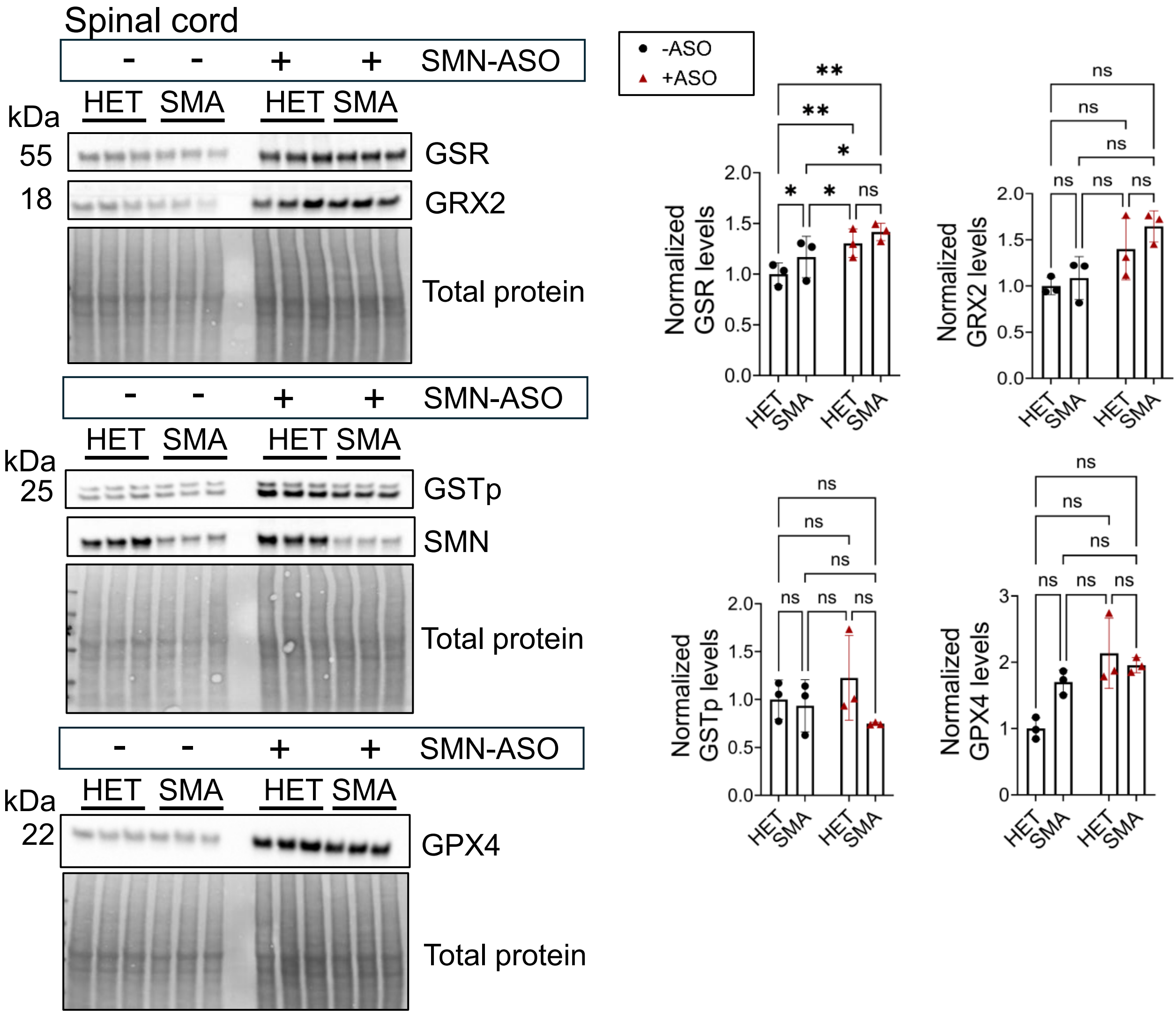

Supplementary Figure S13. Expression of glutathione-related redox enzymes in spinal cord of ASO-treated and untreated SMA and HET mice at postnatal day 10 (P10).

Representative Western blots and quantification of glutathione reductase (GSR), glutaredoxin-2 (GRX2), glutathione-S-transferase p (GSTp) and glutathione peroxidase-4 (GPX4) in spinal cord lysates from HET and SMA mice injected with antisense oligonucleotides (ASO; +ASO) or untreated (-ASO). Blots were normalized to total protein levels. SMA spinal cords showed increased GSR, and ASO treatment led to non significant changes between the ASO groups. SMN-ASO had no significant effect in GSTp, GR2 and GPX4.Two-way ANOVA (factors:genotype and treatment) followed by Tukey‘s post-hoc test for all pairwise comparisons. Bars represent mean ±SD of 3 biological replicates for all groups. Significance thresholds: *p < 0.05, **p < 0.01, ns, not significant.

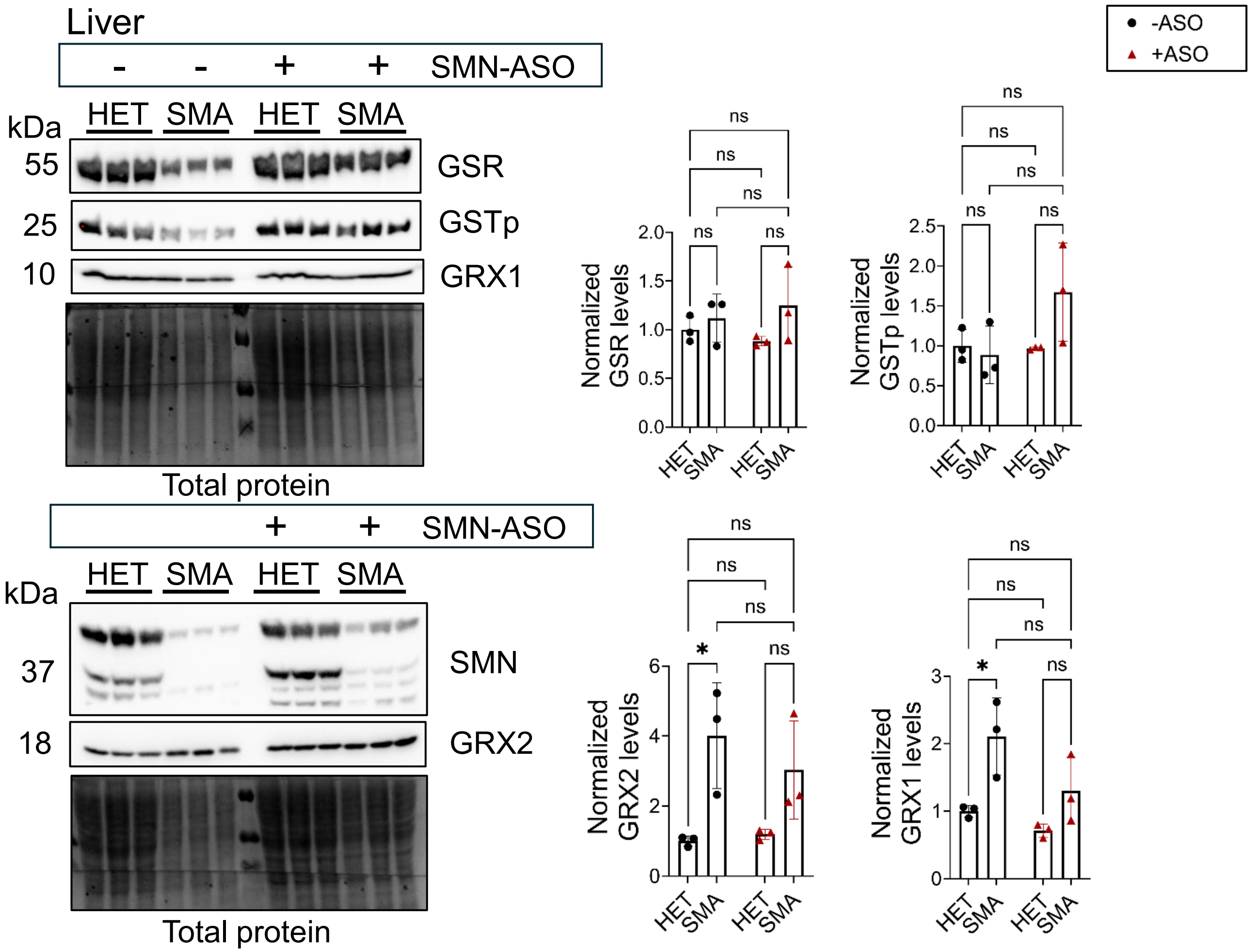

Supplementary Figure S14. Expression of glutathione-related redox enzymes in liver of ASO-treated and untreated SMA and HET mice at postnatal day 10 (P10). Representative Western blots and quantification of glutathione reductase (GSR), glutaredoxin-1 and 2 (GRX1, GRX2), and glutathione-S-transferase p (GSTp) in liver lysates from HET and SMA mice injected with antisense oligonucleotides (ASO; +ASO) or untreated (-ASO). Blots were normalized to total protein. GRX1 and GRX2 were elevated in SMA compared to HET, while ASO treatment reduced this difference, rendering HET+ASO not significantly different, though SMA vs SMA+ASO remained unchanged. All other enzymes showed no significant diffrences across genotypes or treatments.

Two-way ANOVA (factors:genotype and treatment) followed by Tukey‘s post-hoc test for all pairwise comparisons. Bars represent mean ±SD of 3 biological replicates for all groups. Significance thresholds: *$p < 0.05$, ns, not significant.
